# Many-to-one mapping in Mantodea: camouflage strategy and phylogeny drive strike variation in prey capture with raptorial forelegs

**DOI:** 10.1242/jeb.250626

**Published:** 2025-10-13

**Authors:** Lohitashwa Garikipati, Brandon E. Jackson, Christopher E. Oufiero

**Affiliations:** ^1^ Department of Biological Sciences, Towson University, Towson, MD 21252, USA; ^2^ Department of Biological and Environmental Sciences, Longwood University, Farmville, VA 23909, USA

**Keywords:** Praying mantis, Ecomorphology, Feeding behavior, 3D kinematics, Form and function

## Abstract

The evolution of camouflage has led to many examples of organisms mimicking their environment to remain undetected or unseen. Camouflage likely results in strong selective pressures to resemble the environment as it increases Darwinian fitness in both predators and prey, having the potential to result in ecomorphs, which are morphologies that convergently evolve to function in specific environments. Whether the evolution of camouflage in ambush predators results in ecomorphs can be determined by examining the linear morphology and function of the raptorial forelegs among Mantodea (i.e. praying mantises), as the acquisition of food may vary based upon the microhabitat mimicked. We hypothesize that the evolution of camouflage constrains a species' diet based upon available prey in the habitat mimicked, resulting in the evolution of ecomorphs for prey capture. We analyzed over 200 3D high-speed prey capture attempts among ten species, three families and four camouflage strategies. Using principal component analyses to reduce dimensionality of dependent traits and phylogenetic mixed models, our results suggest that the evolution of camouflage does not result in ecomorphs based on raptorial foreleg linear morphology. We also found that camouflage strategy had a significant effect on one kinematic axis, and relatively strong phylogenetic signal but minimal effect of morphology on strike kinematics. Lastly, we recognize two new quantitatively and qualitatively distinguishable hunting strategies in Mantodea. Our results suggest that phylogeny and camouflage shape the versatility of raptorial forelegs in prey capture, which may allow mantises to diversify in their camouflage strategies to exploit different ecological niches, regardless of phylogeny or morphology.

## Teaser

**Summary:** Praying mantis prey capture is versatile and varies interspecifically in velocity and displacement of forelegs, body and starting position, driven by phylogenetic relationships and camouflage strategy.

## INTRODUCTION

Ambush predation has resulted in the evolution of many unique mechanisms of prey capture to quickly minimize the predator–prey gap, including adhesive mechanisms (Anderson and Deban, 2010; Deban and Richardson, 2011; Koerner et al., 2012), elastic mechanisms (Anderson and Deban, 2010; Deban and Richardson, 2011; Longo et al., 2018, 2019), raptorial forelegs (Corrette, 1990; deVries et al., 2012; Kral et al., 2000), camouflage (Pembury Smith and Ruxton, 2020) and speed (Longo et al., 2016). The details of these prey capture mechanisms of ambush predators have been well studied and suggest that there are often multiple ways to be an ambush predator, even within groups. For example, ambush fish predators have been suggested to diversify along an axis of body versus jaw movement. On one axis, camouflaged species, such as a frogfish (*Antennarius hispidus*), remain hidden by being motionless, but use the rapid movement of their mouth to subdue unsuspecting prey, whereas other species such as groupers (*Epinephelus ongus*) remain concealed in burrows and use rapid body movements (Longo et al., 2016). This interspecific comparison among fish suction feeding demonstrates the diversity in the mechanisms of the same prey capture apparatus among ambush predators. However, few have examined prey capture diversity among a particular ambush strategy, such as camouflage specialists.

Camouflage evolution has been a relatively understudied mechanism in the diversity of ambush predator prey capture, despite its importance in minimizing the predator–prey gap (deVries et al., 2012; Pembury Smith and Ruxton, 2020). The evolution of camouflage results in morphological adaptations to avoid detection (masquerading) or remain concealed (crypsis) (Pembury Smith and Ruxton, 2020). Camouflage among ambush predators may allow prey to get closer, potentially increasing prey capture success on a limited subset of species in the microhabitat they are matching (Elliott et al., 1977; Higham et al., 2017; Moore and Biewener, 2015; Oufiero et al., 2024; Shirazi and Higham, 2024). The evolution of similar camouflage strategies among disparate lineages may select for similar patterns of form–function relationships to form ecomorphs. Ecomorphs can be classified by convergent evolution of morphology, probably as a result of adaptations to function in similar environments and microhabitats (Goodman et al., 2008; Losos, 1990; Wainwright and Reilly, 1994). Ecomorphology has direct impacts on organismal fitness, and thus functional variation may indicate fitness variation between the different morphologies among varying microhabitats (Ferry and Higham, 2022; Higham et al., 2021). Recent evidence among more than 200 species of stick and leaf insects (Phasmatodea) has shown convergence in body, head and wing morphology among camouflage strategies into ecomorphs (Boisseau et al., 2025). This highlights the role of camouflage in shaping morphology, suggesting that camouflage diversity among ambush predators may additionally result in functional variation of prey capture.

Mantodea, or praying mantises, are a viable study system to examine questions about the evolution of form–function relationships among camouflaging ambush predators at the order level with few biogeographic constraints. Praying mantises are a group of ∼2500 hypercarnivorous insects characterized by their exaggerated raptorial forelimbs used for prey capture, triangular heads, pseudovein of the forewings and asymmetrical genitalia, among other features (Svenson and Whiting, 2004, 2009). This morphologically diverse group exhibits some of the widest if not the widest array of camouflage strategies in a terrestrial arthropod group and are nearly cosmopolitan in distribution (Svenson and Whiting, 2009; Wieland and Svenson, 2018). Mantises from disparate lineages have evolved similar camouflage strategies in all major continents of the world, and in some cases, repeatedly within several lineages (Svenson and Whiting, 2009; Wieland and Svenson, 2018). Camouflage strategies have also been shown to relate to deviations in foreleg proportions from allometric relations of body size in certain species with some strategies having proportionally longer or shorter forelegs (Oufiero, 2020). The plasticity of arthropod exoskeletons, combined with a strong ecological selective pressure for many different camouflage strategies, has led to the repeated evolution of flower, bark, stick, dead leaf, leaf, grass, moss, lichen, ant, wasp and rock mimicry across the order, tied to occupation and functioning within different microhabitats (Schwarz and Roy, 2019; Svenson and Whiting, 2009; Wieland and Svenson, 2018). These camouflage strategies have been proposed to result in ecomorphs (Oufiero, 2020; Svenson and Whiting, 2009).

Convergence of morphology and function among camouflaging strategies is proposed to be due to selection on aspects of Darwinian fitness, including acquiring resources which can increase reproduction and survival (Wainwright and Reilly, 1994). Praying mantises are well known for their method of prey capture: a rapid extension of their raptorial forelegs to secure the prey with spines, typically from ambush (Corrette, 1990; Loxton and Nicholls, 1979; Maldonado et al., 1967; Oufiero et al., 2016, 2024). Mantises exhibit considerable morphological diversity with respect to their forelegs as noted by several authors (Brannoch et al., 2017; Oufiero, 2020; Rivera and Svenson, 2016). These differences include the length and shape of the forelimbs as well as the spine count and pattern along the foretibia and forefemur (Brannoch et al., 2017; Oufiero, 2020; Wieland and Svenson, 2018). There is also a positive correlation between intraocular distance and foretibia length among species, which affects the depth perception and reach of the mantis, respectively (Oufiero, 2020). Both metrics directly impact the ability of the mantis to capture prey.

The camouflage strategy may limit the types of prey encountered owing to selection for specific microhabitat preferences, resulting in selection on form–function relationships of the raptorial forelegs to function better in the given microhabitat (Pembury Smith and Ruxton, 2020). Species mimicking a particular portion of the micro-habitat may be spending more time in that environment and encounter different prey (Pembury Smith and Ruxton, 2020). For example, the orchid mantis (*Hymenopus coronatus*) which is a flower mimic, tends to feed more on pollinator species, which might require faster movements of the forelegs to capture prey on the fly (O'Hanlon et al., 2015; Svenson et al., 2016). While the details of Mantodea diets are poorly lacking (Prete et al., 2013b; Wilson Rankin et al., 2023), raptorial foreleg form–function relationships may diverge to improve performance and prey capture for different prey in the habitat mimicked (Burnette and Gibb, 2013; Motta et al., 1995; Norton, 1991; Norton and Brainerd, 1993). The raptorial forelimbs of praying mantises are tools used to capture prey, and the morphology of any predator's capture apparatus affects both the ease of capture and potentially the handling time of the prey, which confers a variable fitness compared with other morphologies (Ferry-Graham, 2002). Preliminary evidence from linear measures suggests some camouflage strategies may be adaptive rule breakers in their limb proportions, which in turn might affect the kinematics of prey capture and highlights the need for a thorough comparison of the raptorial foreleg form and function with respect to camouflage to understand ambush prey-capture diversity (Oufiero, 2020).

Prey capture in mantises involves the coordinated movements of three segments of the forelegs during an approach and sweep phase (Copeland and Carlson, 1979; Corrette, 1990; Maldonado et al., 1967; Oufiero et al., 2016; Prete et al., 1990). The general timing of events of the mantis prey capture have been found to be similar across the limited species examined and ontogenetically (Oufiero, 2022; Oufiero et al., 2016). However, these studies have also noted intraspecific variation in prey capture attempts and the resulting kinematics. This variation includes variation in the amount of lunge, foreleg linear and angular velocities, predator–prey positions, and more recently, foreleg lateral displacements (Corrette, 1990; Oufiero, 2022; Oufiero et al., 2016, 2024; Rossoni and Niven, 2020). The use of the raptorial forelegs during prey capture in mantises is based upon studies from limited taxonomic sampling, most often using two-dimensional approaches (Copeland and Carlson, 1979; Corrette, 1990; Maldonado et al., 1967; Oufiero, 2022; Oufiero et al., 2016, 2024; Prete et al., 1990; Rossoni and Niven, 2020). Even with general observation, recent research has highlighted novel convergent hunting strategies, where the forelegs are used to ‘spear’ prey rather than grasp (Rivera and Callohuari, 2020). Furthermore, studies that examine the life history of specific taxa do highlight differences in microhabitat between species and similarities in microhabitat within certain clades, which would impose selection for tools used in locomotion and prey capture, as well as behavior and morphology related to crypsis (Battiston et al., 2020; Garikipati, 2024; Garikipati and Bond, 2021). Therefore, we would expect mantises, like other predator systems, to exhibit diversified prey capture mechanisms, which would provide evidence for the role of camouflage in shaping form–function relationships among camouflage strategies.

This is the first comparative 3D kinematic and morphological exploration of prey capture among mantises specifically and camouflaging ambush predators more generally. We had three goals for this study: (1) to determine whether camouflage strategies result in ecomorphs based on linear morphology of the raptorial forelegs; (2) to characterize the 3D functional space of examined species and camouflage strategy; and (3) to determine whether morphology and camouflage strategy predict function in a phylogenetic framework. We hypothesize that mantis functional morphology will vary among camouflage strategies regardless of phylogenetic relationships, suggesting strong functional ties to camouflage strategies. Alternatively, as this is a comparison across families, we hypothesize that functional morphological differences may also vary based upon phylogenetic relationships, regardless of camouflage strategy.

## MATERIALS AND METHODS

### Sampling and specimen care

**Fig. 1.**
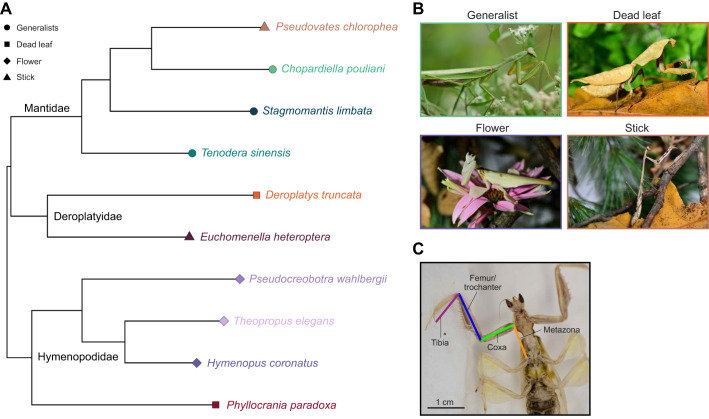
**Evolutionary relationships and camouflage strategies of praying mantis species examined in this study.** (A) Sampled taxa, evolutionary relationships and putative ecomorphs based on camouflage strategy on a pruned phylogeny derived from Svenson and Whiting (2009). Branch lengths are substitutions per site. (B) Examplar mantis species for the four ecomorphs: a generalist (*Tenodera sinensis*; photo credit: R. Turnbaugh), a dead leaf mimic (*Deroplatys truncata*; C. Oufiero), a flower mimic (*Hymenopus coronatus* (M. Wood) and a stick mimic (*Euchomenella heteroptera*; M. Wood). (C) A *Hymenopus coronatus* specimen with the morphology used in this study. Orange line represents metazona length, the distance between points 1 and 5 from the videos; green line represents coxa length, distance between points 1 and 2; blue line represents femur length, distance between points 2 and 3; and purple line represents tibia length, distance between points 3 and 4.

To investigate variation in prey capture strategies by camouflage, we obtained 3–5 females from 10 different species from domestic hobbyists (Fig. 1). These species represented four different camouflage strategies: (1) ‘generalist’ (*Tenodera sinensis*, *Chopardiella pouliani*, *Stagmomantis limbata*); (2) ‘dead leaf’ (*Deroplatys truncata*, *Phyllocrania paradoxa*); (3) ‘flower’ (*Hymenopus coronatus*, *Pseudocreobotra wahlbergi*, *Theopropus elegans*); (4) ‘stick’ (*Euchomenella heteroptera*, *Pseudovates chlorophaea*) (Fig. 1B; see Table S1 for taxonomic information, which follows the system developed by Schwarz and Roy (2019). We classified camouflage purely by apparent cryptic features or lack thereof, similarly to other studies. For example, generalists were considered to be mantises that lacked large projections on their legs, abdomen and pronotum and do not clearly match any particular part of the microhabitat; flower mimics were species that were white, yellow and green with lobed legs; stick mimics were elongate species with brownish/gray and mottled coloration; and dead leaf mimics were primarily brown to black with lateral pronotal projections and leg lobes (Svenson and Whiting, 2009; Wieland and Svenson, 2018). While these categorizations are somewhat subjective and broad, for a cursory examination of diversity and variation, we feel that these groupings suffice and are consistent with others (Svenson and Whiting, 2009; Wieland and Svenson, 2018).

The ten species represent taxa from three different families (Mantidae, Hymenopodidae and Deroplatyidae) from most major regions of the world (Neotropical, Afrotropical, Oriental and Nearctic regions). Nymphs were reared to adulthood in 12.7×12.7×17.8 cm clear containers with fabric gauze on the top and sides for mantis to climb freely. All species were fed to satiation every 3 days as nymphs and only fed on practice or trial days as adults to promote a strong feeding response during trials. Mantises were fed *Drosophila melanogaster* and *Drosophila hydei* when young and *Blatta lateralis* as older juveniles. All mantises were provided water every 3 days using a spray bottle. The vivarium room was kept on a 12 h:12 h light:dark cycle, at 24.3±0.0031°C (mean±s.e.m.) and 48.12±0.094% relative humidity throughout the study. All species were housed with USDA permission onsite at Towson University in the Science Complex Vivarium (permit no. P526P-22-06241).

### 3D filming

For each trial, mantises were placed upside down on a feeding platform and allowed to settle, determined by when the mantis stopped moving and began to groom. Filming was conducted in the same room where adult mantises were housed, therefore the temperature and humidity were the same as rearing conditions. Two Edgertronic SC-1 cameras (Sanstreak Co. San Jose, CA, USA), placed to record ventrally and laterally, were used to record the prey capture attempts of the mantises (1000 frames per second with a shutter speed of 50 µs). Prior to each trial, mantises were starved for a minimum of 2 days, and up to 5 days, to ensure motivated strikes. Mantises were illuminated with a Skylux Wescott light and four CM Vision 850 nm IR lights (Fig. S1, Movies 1–5).

To elicit a feeding response, a blue bottle fly (*Calliphora vomitoria*) was attached to a fishing line, and waved in a random fashion in front of the mantis to represent evasive, semi-natural prey, as in previous studies (Oufiero, 2022; Oufiero et al., 2016, 2024). The rationale for this method was to ensure each mantis was striking when the prey item satisfied its threshold for a potentially successful strike. We felt that standardizing the location of prey for each strike would narrow the observed variation in prey capture behavior under semi-natural conditions if a feeding response could even be elicited. Furthermore, as these species vary in their interocular distance and potential depth perception, a standardized location might not be representative for each species. Subsequent trial attempts only occurred after the mantis had finished consuming the captured fly and cleaned, signaling a completed meal. Owing to the size variation in examined species and satiation thresholds, some species were tested over several days or weeks. In total, we attempted to collect five trials (minimum of three) for each of the 48 individuals, resulting in 239 prey captures in total (see Movies 1–4 for examples).

### Kinematic data preparation

Ten body points on videos from both cameras were tracked with a combination of machine learning and manual tracking to obtain a set of 3D kinematics and behaviors similar to previous studies using 2D (Oufiero, 2022; Oufiero et al., 2016) and manual 3D (Oufiero et al., 2024) (see Fig. S2).

We trained two machine models on the lateral and ventral views in DeepLabCut (v. 2.2.1.1; Mathis et al., 2018; Nath et al., 2019), one for mantises that tend to keep their coxae partially abducted so that the starting coxa angle is greater (e.g. 50 deg or more), such as *Hymenopus coronatus*, and one for where the coxa is not abducted and the coxa start angle is lower (<50 deg, see Results). For each model, 12 strikes, including four species per lower starting coxa angle model and only *H. coronatus* for the model for higher starting coxa angles, were fully manually digitized in DLTdv8 (Hedrick, 2008). The DLTdv8 coordinates for all frames were converted to DeepLabCut format using custom code (dlc2dltlabels function in DLCConverterDLT, available at https://github.com/backyardbiomech/DLCconverterDLT). All frames were included in the DeepLabCut models, for a total of 138.75±36.44 (mean±s.d.) frames taken from 12 individuals for the lower starting coxa model and 57.25±18.99 frames taken from 12 individuals for the greater coxa starting model. Of those frames, 95% were used to create the training data set, with 5% held aside for testing. We used a Resnet-50 based neural network with default parameters for both models. The greater coxa starting model was trained for a total of 2 million iterations, evaluated to test error of 2.68 pixels and train error of 2.18 pixels. The lower starting coxa model was trained for 1.7 million iterations with a test error of 4.13 pixels and train error of 2.45 pixels. We used a *p*-cutoff of 0.6 to estimate body part coordinates when analyzing other videos from similar body positions.

In each machine model, 10 points on each view were tracked (Fig. S2). Points 1–4 were placed on the left leg: point 1 at the joint where thorax connects with the coxa, point 2 at the trochanter, point 3 where the femur meets the tibia and point 4 where the tibia ends. Point 5 at the joint where the front leg connects to the thorax, point 6 on the prey, and point 7 at the center of the left eye. Points 8–10 were placed on the right leg: point 8 at the trochanter, point 9 where the femur meets the tibia, and point 10 where the tibia ends.

3D reconstruction of tracked points was performed in DLTdv8. We recorded a 3D calibration structure, an initial one used on a small number of trials including 12 points filling a volume approximately 9×5×8 cm for the first 8 days of filming. We then switched to a calibration structure including 15 points constructed of Lego structure filling a volume approx. 12×8×9 cm. DLT coefficients were determined in DLTdv8, returning reconstruction errors of 1.56±0.559 (mean±s.d.) for the 12-point calibration across 8 days of filming and 7.43±0.839 (mean±s.d.) for the 15 point calibration across 27 days of filming. After the DeepLabCut machine models were used to analyze each video, we used custom script (*dlc2dlt* function in DLCConverterDLT) to convert the tracked coordinates from DeepLabCut into DLTdv8, where errant tracking from DeepLabCut was manually edited (Movie 5). This resulted in a max reconstruction error of 2.288 pixels for the 12-point calibration and 9.348 pixels for the 15-point calibration across all trials.

The 3D coordinates were then run through custom R script that produces a suite of 3D kinematic variables for each attempt, including predator–prey distance and angle, approach and sweep times, angular and linear velocities of each foreleg segment and lateral displacement (Oufiero et al., 2024). The start of the strike was defined as the moment the mantis moved its forelegs and the end of the strike was defined as the point the prey item crossed into a triangle formed by the forelegs, representing the moment of prey capture (Oufiero, 2022; Oufiero et al., 2016). A tenth order polynomial was used to smooth out the data to correct for human error similarly to previous studies (deVries et al., 2012; Oufiero, 2022; Oufiero et al., 2016, 2024). From the smoothed data we obtained standardized angles of all joints during the strike as the angle of each joint at a given point in time minus the minimum angle of each joint. The maximum coxa angle was 99% of the maximum measured angle (e.g. Oufiero et al., 2024). For the femur and tibia, the maximum angles were gathered with the findpeaks function (pracma package in R, v2.4.2; https://CRAN.R-project.org/package=pracma). Both segments open during the approach and then close during the sweep, resulting in an angular kinematic peak. As a result, the standardized, smoothed angle at that peak represented the maximum trochanter/femur and tibia angles (Oufiero et al., 2024). Angular velocities were calculated as the derivative of the smoothed angle over time, and maximums were the maximum value of each. For the tibia, which must close quickly to secure the prey, the minimum velocity (a negative value) represented the maximum when flexing, so we used the absolute value of the lowest minimum tibia velocity. We also obtained the linear displacement of each foreleg segment to obtain the linear velocity of each (Oufiero, 2022). Body velocity was calculated based on the movement of a point representing the mean between the procoxal joint and the mesocoxal joint (points 1 and 5). Subsequently, body displacement was smoothed using a 10th order polynomial, and the derivative of the line over time was used to calculate velocity. Approach and sweep time were defined similar to previous studies, with the approach beginning when the coxa is at 5% of the peak coxa angle and ending when the trochanter/femur velocity was at 10% of its maximum (Corrette, 1990; Oufiero, 2022; Oufiero et al., 2024). The end of the approach also signifies the beginning of the sweep which continues till prey capture (Corrette, 1990; Oufiero, 2022; Oufiero et al., 2016). We also obtained the lateral displacement of the coxa/trochanter, femur/tibia and tip of the tibia. These were obtained by taking the maximum, smoothed displacement between each joint on the left and right forelegs (Oufiero et al., 2024). These variables were then used in subsequent analyses.

In our final data set we included the lateral displacements, linear and angular velocities of the coxa, femur, tibia, as well as the coxa starting angle, approach and sweep time, predator prey angle and distance at the start of the strike, body displacement (i.e. lunge) and velocity for a total of 16 kinematic variables (Table S2). We also obtained morphology from the videos (Oufiero, 2022). Our morphological data set included lengths of the forecoxa (distance from point 1 to point 2), forefemur (distance from points 2–3), and foretibia (distance from points 3–4), as well as the length of the metazona (length from the coxa to the posteroventral margin of the propleura/anterior of the mesocoxa, points 1–5, Fig. 1C, Table S3). Each of these segments was averaged throughout the strike (Oufiero, 2022).

### Statistical analyses

The kinematic data was analyzed in RStudio v. 4.3.1 (r-project.org). From 239 total attempts, we first extracted the three fastest strikes per individual (total 144) based on tibia and femur angular velocities to represent maximally motivated strikes (Oufiero et al., 2016). We ran a principal component analysis (PCA) for the 16 kinematic variables (Table S2). We ran a separate PCA on our four morphological traits (Fig. 1, Table S3). Each PCA was followed by a broken stick model to determine which principal components significantly contributed to observed variation (factoextra, https://CRAN.R-project.org/package=factoextra; vegan, https://CRAN.R-project.org/package=vegan).

We first explored the functional space for all maximally motivated attempts across individuals and species (144 prey capture attempts). Then, we obtained the mean of the three maximally motivated attempts for each individual for a phylogenetic generalized linear mixed model (PGLMM), resulting in 48 individuals across 10 species (De Villemereuil and Nakagawa, 2014; Whitlow et al., 2019). A pruned phylogeny developed by Svenson and Whiting in 2009 was used to correct for phylogenetic relationships. If the species examined in our study was not present in the phylogeny, we used another species in the same genus, if not, then tribe and if not, then subfamily (Fig. 1). For *Chopardiella pouliani* (Mantidae: Vatinae: Heterovatini) the closest outgroup taxon available was *Oxyopsis* sp. *#*MN294 (Mantidae: Stagmatopterinae: Oxyopsidini); since we sampled *P. chlorophaea* (Vatinae: Vatini) we wanted to avoid selecting taxa that were too closely related.

We analyzed a series of PGLMMs for each significant morphological and functional PC axis (De Villemereuil and Nakagawa, 2014; Whitlow et al., 2019). First, we examined whether morphology of the forelegs varies among camouflage strategies using a PGLMM. Two models were analyzed, one for each significant morphological PC axis (see below), with camouflage strategy as a fixed effect (four levels: flower, stick, dead leaf and generalist) and the random effect of phylogeny. We next analyzed a series of PGLMM models for the significant functional PC axes (see below). For each PGLMM, the independent continuous traits (morphology PC axes 1 and 2) were included as species means and intraspecific variation was calculated as the difference between each species mean trait value and each individual's value for a given species as outlined in De Villemereuil and Nakagawa (2014). We included the fixed effect of camouflage strategy (generalist, flower, dead leaf and stick). If there was no significant effect of morphology, we removed those continuous traits and examined a model with only camouflage. We also compared the AICc of models with camouflage and morphology or just camouflage when morphology wasn't significant to determine if it was better fit. Each PGLMM was run 1,000,000 times and included the random effect of the phylogeny and species (Whitlow et al., 2019). PGLMMs are like other phylogenetic comparative methods (e.g. phylogenetic independent contrasts or phylogenetic generalized least squares) in that the phylogeny is included as a covariance matrix, in this case as a random effect in the mixed model (De Villemereuil and Nakagawa, 2014). The difference is that in PGLMMs, multiple individuals per species can be included, as opposed to analyzing species means, incorporating the within species variance. We also followed with a *post hoc* examination using emmeans (https://CRAN.R-project.org/package=emmeans) to examine the pairwise differences among the habitat mimics. Lastly, we estimated lambda for each PGLMM model, a measure of phylogenetic signal (De Villemereuil and Nakagawa, 2014). Phylogenetic signal tests for the similarity in traits or relationships among closely related species (Blomberg et al., 2003). If there are no significant predictors of our dependent trait and a strong phylogenetic signal for the model, it suggests that phylogenetic relationships are driving the variation in dependent traits. The raw kinematic data (Dataset 1) used for this study and the R script (Dataset 2) are provided as supplemental files, along with the output of the R Markdown file (Dataset 3).

## RESULTS

### Examining morphology with phylogeny

**Fig. 2.**
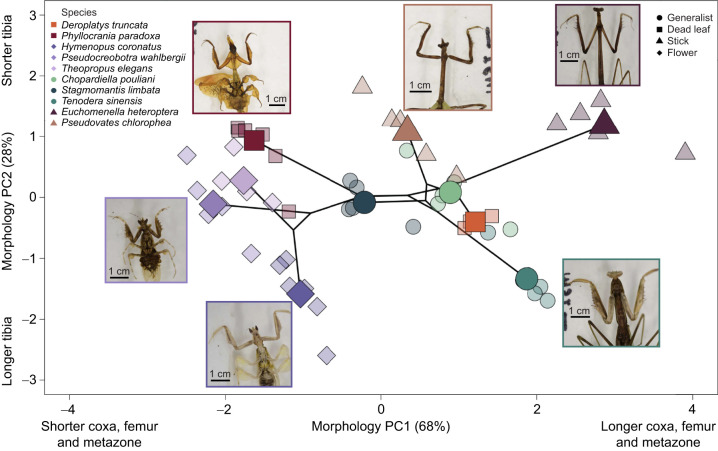
**Morphospace of examined praying mantis species. Individual (smaller points), species means (larger points), phylogeny (solid black lines) and representative specimen photos are shown.** Phylogeny appears to largely drive body lengths as there was no significant effect of camouflage on morphology PC1 (*P*>0.05) or PC2 (*P*>0.05).

A broken stick model revealed two significant PC axes for linear morphology (Fig. 2). PC1 (68.00% of the variation) describes longer coxa, femur and metazona lengths. PC2 (28.72% of the variation) describes longer tibia lengths. Based on the PGLMM there was no significant effect of camouflage strategy on PC1 (*P*>0.05) or PC2 (*P*>0.05). Furthermore, *post hoc* comparisons using the emmeans function in R revealed no specific differences among habitat mimics, suggesting that phylogeny is largely driving segment length variation (Fig. 2). Flower mimics in the Hymenopodidae tend to have the smallest metazone lengths (*H. coronatus*=0.852±0.041 cm; *P. wahlbergii*=0.795±0.41 cm; *T. elegans*=0.961±0.041 cm; means±s.e.m.) compared with the stick mimics from the other families (*E. heteroptera*=4.369±0.066 cm; *P. chlorophea*=3.172±0.070 cm) Table S3). Proportion of foreleg length tends to vary among species, for example *T. sinensis* has long coxa (1.808±0.028 cm), femur (2.52±0.044 cm) and tibia lengths (1.05±0.015 cm), whereas *E. heteroptera* has long coxa (1.91±0.023 cm), and femurs (2.67±0.022 cm) but short tibias (0.744±0.020 cm, Table S3). Finally, the PGLMM model with morphology PC1 as the dependent had a lambda of 0.6889 and the morphology PC2 as the dependent trait had a lambda of 0.4906, suggesting moderate to strong phylogenetic signal.

### Characterizing 3D functional space of examined species and camouflage strategies

**Fig. 3.**
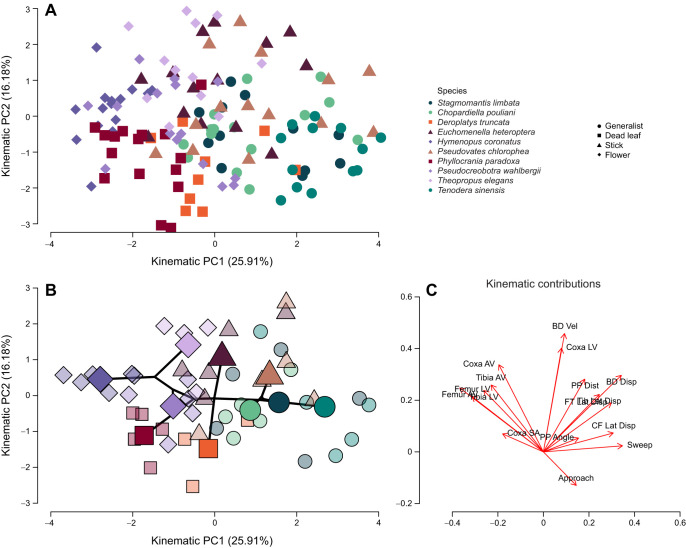
**Functional space of prey capture kinematics in mantis species based on PC1 and PC2.** All analyzed prey captures (A, *N*=144) and phylo-functional space (B) of individual (small points, *N*=48) and species means (large points, *N*=10), including phylogenetic relationships from Fig. 1A (solid black line). (C) Individual kinematic contributions (e.g. loadings) to each PC axis. PC1 (25.91%) describes attempts incorporating more lunge and lateral displacement, as seen in some generalist and stick camouflage strategies in the Mantidae, which trades off against femur and tibia speeds, as seen in Hymenopodidae. PC2 (16.18%) describes faster coxa and body movements which trade off against longer approach times, which the dead leaf camouflage strategies have converged upon in the two families (Hymenopodidae and Deroplatyidae). Kinematic contributions as represented by the loadings of each of the 16 kinematic variables on PC1 and PC2. AV, angular velocity; LV, linear velocity; SA, starting angle; Lat. Disp., lateral displacement; Disp., displacement.

**Fig. 4.**
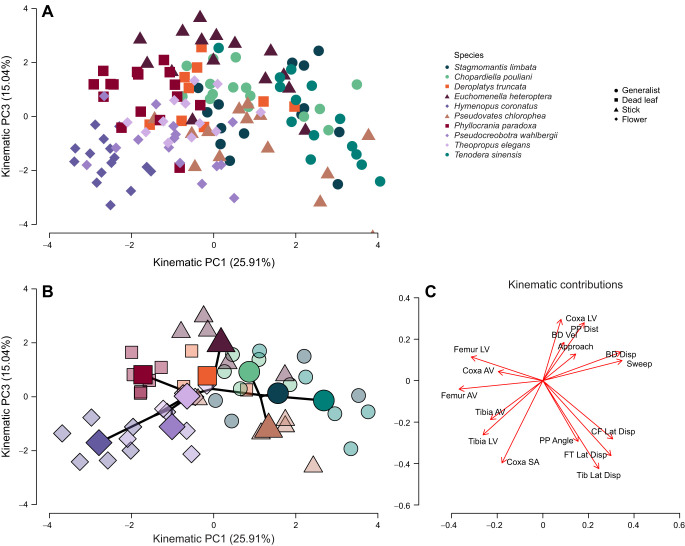
**Functional space of prey capture kinematics in mantis species based on PC1 and PC3.** All analyzed prey captures (A, *N*=144) and a phylo-functional space (B) of individual (smaller points, *N*=48) and species means (large points, *N*=10), including phylogenetic relationships from Fig. 1 (solid black line). (C) Individual kinematic contributions (e.g. loadings) to each PC axis. PC1 (25.91%) is the same axis as PC1 in Fig. 3. PC3 (15.04%) characterizes species with anterograde prey capture, which tend to start with their coxa at a greater angle (low PC3) as exemplified by *H. coronatus* and *P. wahlbergi* both flower mimics in Hymenopodidae and *P. chlorophaea* a stick mimic in Mantidae. Anterograde is also characterized by relatively wider lateral displacements, as exemplified by *P. chlorophaea* and faster tibia flexion, exemplified by *H. coronatus*. This strategy trades off with some basigrade hunters that attempt to capture prey farther away with greater body and coxa velocity as exemplified by the stick mimic *E. heteroptera* (high PC3).

We visualized the first interspecific 3D functional space of praying mantises with our three maximally motivated strikes for each individual (Figs 3A and 4A). The broken stick model on all maximally motivated attempts (*N*=144) resulted in three significant PC axes for prey capture kinematics (Table 1). Kinematic PC1 (kPC1) (25.91% variation) describes strikes that use greater lunge (body displacement), greater lateral displacements of all segments, and longer sweep times trading off against strikes that had faster femur and tibia velocities (angular and linear Fig. 3A). kPC2 (16.18% variation) described faster coxa and body movements, which trade off against approach times. Lastly, kPC3 (15.04%) described attempts on prey farther away with high coxa linear and body velocities trading off against those that were on prey at a greater angle and had a greater coxa starting angle, faster tibia linear and angular velocity, and greater lateral displacement of segments (Table 1).

**Table 1.** Principal component analysis loading results of the 16 kinematic variables obtained from 144 prey capture attempts in praying mantises

	PC1	PC2	PC3	PC4	PC5	PC6	PC7	PC8	PC9	PC10	PC11	PC12	PC13	PC14	PC15	PC16
Coxa LV	0.0797	0.4019	0.2960	0.1797	0.0535	0.0672	0.2976	0.4471	0.0070	0.4490	0.0411	0.2696	0.2826	0.1574	0.1598	0.1009
Femur LV	−0.3140	0.2147	0.1147	0.1701	−0.3685	−0.3104	−0.0235	0.3286	0.0010	−0.2427	−0.1545	−0.1700	0.0031	0.1002	−0.5801	0.1197
Coxa AV	−0.1956	0.3375	0.0441	0.0692	0.5019	0.3103	−0.0732	0.0980	−0.0523	−0.3523	0.1908	0.3496	−0.3706	−0.1114	−0.2024	−0.0414
Femur AV	−0.3676	0.2478	−0.0407	0.2665	−0.1564	0.0692	−0.1087	−0.0425	0.1170	−0.3727	−0.2205	−0.0561	0.1255	0.1796	0.6192	−0.2283
Tibia AV	−0.2296	0.2591	−0.1878	−0.3734	0.0749	−0.0540	0.4531	−0.2735	−0.3212	−0.1595	0.1013	−0.0139	0.4968	−0.1058	−0.1090	−0.0886
Tibia LV	−0.2624	0.2380	−0.2618	−0.2487	−0.1876	−0.0743	0.3640	−0.0981	0.3870	0.2949	0.1130	−0.0603	−0.5403	0.1044	0.0723	−0.0083
Coxa start angle	−0.1792	0.0698	−0.3970	−0.1117	−0.3618	0.1304	−0.4861	−0.0392	−0.0264	0.2099	0.0935	0.5414	0.1885	−0.0154	−0.0754	0.1381
Approach time	0.1444	−0.1297	0.1287	0.3740	−0.4817	0.2848	0.4181	−0.3021	−0.2781	−0.1380	0.0283	0.2873	−0.2056	−0.0005	−0.0522	0.0436
PP angle	0.1546	0.0536	−0.2933	−0.1196	−0.1590	0.7262	0.0490	0.3548	0.0238	−0.0393	−0.0991	−0.4007	0.0672	−0.0471	−0.1026	−0.0464
PP dist.	0.1816	0.2817	0.2800	−0.3062	−0.3548	−0.1423	−0.1931	0.1819	−0.2916	−0.0695	0.2658	−0.0913	−0.2079	−0.4384	0.2565	−0.1699
Body vel.	0.0929	0.4582	0.1848	0.2046	0.0148	0.1391	−0.1782	−0.5191	0.2492	0.1964	−0.1913	−0.2183	0.1011	−0.3611	−0.1428	0.1968
Body disp.	0.3422	0.2965	0.1376	−0.2183	−0.0455	0.0547	−0.2142	−0.2496	−0.0989	−0.0755	0.1262	−0.0749	−0.0407	0.7425	−0.1192	−0.1047
Sweep time	0.3474	0.0235	0.0970	−0.3851	−0.0910	−0.0487	0.1497	0.0719	0.4584	−0.3902	−0.3878	0.3780	0.0729	−0.0873	0.0117	0.1189
Coxa/femur LD	0.3068	0.0733	−0.2813	0.3298	−0.0564	−0.1644	0.0622	0.0410	0.3887	−0.2427	0.6410	−0.0516	0.2144	−0.0672	−0.0266	−0.0460
Femur/tibia LD	0.2990	0.1901	−0.3620	0.2102	0.0591	−0.2190	0.0205	0.0351	−0.1295	0.1560	−0.3691	0.1339	−0.0949	−0.0931	−0.1577	−0.6390
Tibia LD	0.2457	0.2225	−0.4248	0.1024	0.1133	−0.2100	0.0277	0.0685	−0.3422	−0.1218	−0.1625	−0.0883	−0.1749	0.0355	0.2266	0.6225
Proportion variance	0.2591	0.1618	0.1504	0.0952	0.0779	0.0617	0.0477	0.0335	0.0251	0.0226	0.0172	0.0144	0.0118	0.0091	0.0067	0.0058
Cumulative variance	0.2591	0.4209	0.5713	0.6665	0.7444	0.8061	0.8538	0.8873	0.9124	0.9350	0.9522	0.9666	0.9784	0.9875	0.9942	1.0000

LV, linear velocity; AV, angular velocity; LD, lateral displacement. Proportional and cumulative variance of each PC axis included. A broken stick model suggests that only PC axes 1–3 explain a significant amount of variation.

### Examining functional morphology with phylogeny

Using individual means and phylogeny in a PGLMM, there was no significant effect of morphology or camouflage after accounting for phylogeny on kPC1 (25.91%, *P*>0.05, Fig. 3B). The differences in the velocity of the femur and tibia versus the amount of lunge and lateral displacement seems to be driven by phylogenetic relationships. The Hymenopodidae tend to have increased velocity of the femur and tibia regardless of camouflage (flower and dead leaf, Fig. S3). For example, *H. coronatus* had an average angular velocity of the femur of 9258.7±370.7 deg s^−1^ (mean±s.e.m.) and a tibia angular velocity of 14,140.0±851.2 deg s^−1^, whereas *T. sinensis* had a femur angular velocity of 4045.3±668.9 deg s^−1^ and a tibial angular velocity of 7650.5±801.5 deg s^−1^, an increase of ∼129% for the femur and ∼85% for the tibia. Most of the Mantidae tend to use more lunge and lateral displacement of the forelegs (Table S2). For example, *T. sinensis* had an average body displacement of 1.33±0.14 cm, whereas *H. coronatus* has an average body displacement of 0.38±0.05 cm, an increase of 251% body displacement. The Deroplatyidae (*D. truncata* and *E. heteroptera*) are in the middle, diversifying more on kPC2 (Fig. 3B). The model with morphology and camouflage had a lambda of 0.5098, suggesting moderate amount of phylogenetic signal. The model with just camouflage was better fit (AICc=61.38 vs AICc=152.46 for full model), with a higher phylogenetic signal (lambda=0.7006), but there was still no significant difference among camouflage strategies (*P*>0.05).

The full model, including morphology PC1 and PC2 as well as camouflage, resulted in no significant effect of morphology and a marginal effect of camouflage on kinematic PC2 with a lambda of 0.4003. However, this model (AICc=159.01) was not as well supported as a model with just camouflage (AICc=150.73), which showed a significant effect of camouflage and had a lambda of 0.3311. *Post hoc* pairwise comparisons of the model with just camouflage revealed that dead leaf mimics were marginally significantly different than flower mimics (*P*=0.0495) and stick mimics (*P*=0.0132), tending to have longer relative approach times, shorter prey distances, and slower coxa linear and body velocities (Table S2, Fig. 3B). For example, *D. truncata* had approach times of 0.031±0.0061 s compared with *H. coronatus* (flower) and *E. heteroptera* (stick), which had approach times of 0.011±0.0008 and 0.016±0.0031 s, respectively (means±s.e.m.). Conversely, *D. truncata* had a coxa velocity of 69.3±3.06 cm s^−1^ and a body velocity of 32.4±5.10 cm s^−1^, whereas *T. elegans* (flower) had coxa linear and body velocities of 84.2±4.37 cm s^−1^ and 63.3±6.11 cm s^−1^ and *E. heteroptera* (stick) had coxa and body velocities of and 116.7±3.30 cm s^−1^ and 52.3±4.55 cm s^−1^ (Fig. S4, Table S2).

**Fig. 5.**
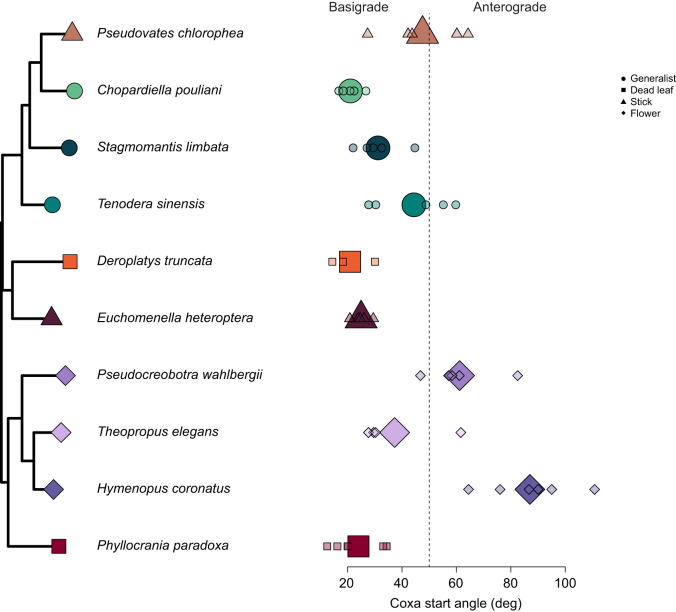
**Starting angle of the coxa joint among species of praying mantis examined in this study.** Larger points represent species means; smaller points represent individual means. Shapes and colors represent different camouflage strategies. We identify a starting angle of 50 deg as separating basigrade (<50 deg) and anterograde (>50 deg) hunters. This starting angle has a strong loading on kinematic PC3.

The full model for the third kinematic PC axis (kPC3, 15.04%) had a significant effect of species morphology PC1 (ß=0.8871, *P*=0.010) and species morphology PC2 (β=1.4181, *P*=0.006). Species with longer coxa and femurs tended to have faster coxa linear velocities, whereas species with longer tibia tended to initiate strikes at higher coxal angles (Fig. S5). *Post hoc* comparisons of camouflage showed a significant difference between stick mimics with dead leaf mimics (0.0154) and generalists (*P*=0.0285), but no other mimics differed significantly (*P*>0.05) (Fig. 4). This model had a lambda of 0.3834. However, this model was not as well supported as the model with just camouflage (full model AICc=151.38, camouflage only model AICc=139.24), which showed no significant difference among camouflage strategies and had a higher phylogenetic signal (lambda=0.5552). Based on this kinematic axis, we identify two hunting strategies in Mantodea. Some species initiate their strikes with high coxa starting angles compared with other species. For example, two flower mimics (*H. coronatus* and *P. wahlbergii*) initiate prey capture attempts with their coxa at greater than 50 deg (*H. coronatus*: 87.02±5.76 deg; *P. wahlbergii*: 61.18±4.54 deg; Fig. 5).

## DISCUSSION

The goals of this study were to assess the role of camouflage evolution on form–function relationships of praying mantis raptorial forelegs, incorporating phylogenetic relations. We found that the evolution of similar camouflage strategies did not result in the convergence of foreleg linear morphology in our sample (Fig. 2). While stick mimics share similar relative tibia lengths (high morphology PC2), it is not significantly different from other camouflage strategies; for example, the dead leaf mimic *P. paradoxa* exhibits similar foreleg lengths (Fig. 2). The relatively high phylogenetic signal for each axis (morphology PC1 lambda=0.6889, morphology PC2 lambda=0.4906) suggests that phylogeny, and not camouflage strategy, appears to drive the observed distribution in linear morphology of our sample, which is in agreement with larger interspecific comparisons on linear traits (Oufiero, 2020). This is in contrast to recent studies of Phasmatodea, which show that the evolution of camouflage results in ecomorphs (Boisseau et al., 2025). The discrepancy may be in the limited morphological sampling in our study that is not fully capturing foreleg or ecomorph variation. Expanding the breadth of studies to include additional morphological features may demonstrate camouflage strategy resulting in ecomorphs among Mantodea (Boisseau et al., 2025; Brannoch et al., 2017; McLean et al., 2021).

A defining feature of an ecomorph is morphological convergence to function within the habitat niche (Losos et al., 1998; Wainwright and Reilly, 1994; Williams, 1983). Mantises have been proposed to fall into ecomorphs based upon camouflage strategies (Svenson and Whiting, 2009; Wieland and Svenson, 2018), but there has yet to be a study categorizing morphology related to mimicry outside of linear measures of the limbs and body (Oufiero, 2020). Although there are limitations to linear morphology of the raptorial forelegs alone, which might not be capturing the phenotypic variation of the complex raptorial forelegs or rest of the morphology (Boisseau et al., 2025; McLean et al., 2021), the results in this study imply that intrafamilial similarity in limb proportions does not necessarily constrain species from evolving to mimic different parts of the environment, allowing them to avoid predation and where they potentially may encounter different types of prey and thus select for variable kinematics of the strikes. Mantis lineages appear to exhibit adaptive radiation, for example, the Dominican Epaphroditidae evolved bark, dead leaf and generalist mimicry strategies from a presumed generalist ancestor (Svenson and Rodrigues, 2017). Adaptive radiation in Mantodea may be abundant owing to the flexibility of raptorial forelegs; our results show that, even with little modification of the segment lengths, the forelegs can be used in different ways to capture prey (Figs 3 and 4), allowing mantises to quickly diversify as they expand or alter their niche.

The versatility of the raptorial forelegs for prey capture is highlighted in our functional spaces (Figs 3 and 4), building upon prior intraspecific studies (Oufiero et al., 2016, 2024; Rossoni and Niven, 2020) and similar to interspecific studies in other groups (McLean et al., 2024). The versatility of the forelegs may be due in part to their flexibility – both the forecoxa and forefemur-trochanter are able to adduct/abduct, flex/extend and can rotate on the condyle (Corrette, 1990; Gray and Mill, 1983). This flexibility likely relates to observed kinematic variation. Intraspecific results from 2D analyses have revealed non-stereotypic prey capture attempts in mantises that can vary in relation to prey speed, prey distance, and through ontogeny (Oufiero, 2022; Oufiero et al., 2024; Rossoni and Niven, 2020). Our functional space highlights both intra- and inter-specific flexibility in movements to capture prey (Figs 3 and 4) and may correlate to dietary differences among families and camouflage strategies in their natural habitats.

Examination of the functional space (Figs 3 and 4) shows that kPC1 (25.91% variation) describes strikes that incorporate greater lunge and foreleg lateral displacement (high kPC1), which trade off against faster segment movements and thus faster prey capture attempts (low kPC1). Similarly to morphology, these differences appear to be driven by phylogeny (lambda=0.5098) as there is no significant effect of camouflage or morphology, which suggests different lineages use more or less lunge and speed irrespective of camouflage. The examined Hymenopodids tended to have faster femur and tibia movement and incorporate less lunge, whereas the Mantids incorporated more lunge, representing a trade-off between relying on the prey to come into range versus moving into range to capture prey (i.e. sedentary vs active, Fig. S3). This is similar to a main axis of ambush predator diversity: (1) among fish, which can be sedentary with lots of jaw movement versus using body ram to close the predator–prey gap (Longo et al., 2016); and (2) in Amblypygi species that vary in the amount of body used in relation to raptorial pedipalp lengths (McLean et al., 2024). The increased angular velocity of the tibia and femur in Hymenopodids is potentially the result of increased contraction speeds of the muscles, suggesting adaptations to capture faster prey, which is different from that found ontogenetically (Oufiero, 2022). The lack of an effect of morphology and camouflage strategy on kPC1 suggests a potential many-to-one system, with multiple phenotypic traits (kinematics) affecting one performance, prey capture (Bergmann and McElroy, 2014; Wainwright, 2005). This also suggests that phylogenetic groups are diversifying in the speed versus the amount of lunge and lateral displacement used, but diversifying into habitat mimics leads to changes in kinematics along the second main axis of kinematic variation.

The second axis of kinematic variation (kPC2: 16.18% variation) described prey capture attempts that varied in their coxa and body speed versus approach time. On this functional axis, dead leaf mimics have significantly slower coxa and body movements and longer approach times than flower- and stick-mimicking species (Fig. S4). The approach phase of a mantis prey capture attempt is characterized by the abduction of the coxa (Corrette, 1990; Oufiero et al., 2016), so a longer approach time should trade off against a slower coxal velocity, as our results indeed show. Longer approach phases have also been shown to result in a greater probability of prey capture success in *Sphodromantis lineola*, which may improve accuracy in dead leaf mimics or allow them to remain undetected for longer (Oufiero et al., 2024; Pembury Smith and Ruxton, 2020). However, the selection pressures for this improved accuracy in a dead leaf-mimicking environment remain to be examined. The other mimics (stick, flower and generalists) exhibited similar kinematics along PC2 despite their different phylogenetic relations, again suggesting they are diversifying more along other axes. To our knowledge, this is the first example of camouflage strategy affecting prey capture among ambush predators. Further exploration of the role of camouflage in prey capture, particularly in natural settings, would provide further evidence of its role to determine if there are consistent principles.

In the best fit model, the last significant kinematic axis (kPC3 15.04% variation) was not influenced by morphology or camouflage but described prey capture attempts that suggest a trade-off in the starting coxa angle and lateral displacement with the coxa linear velocity and prey distance (Figs 4 and 5). We characterize these two prey capture strategies as anterograde (high starting coxa angle >50 deg, low kPC3) and basigrade (low coxa starting angle <50 deg, high kPC3) strikes, that are visually recognizable and occupy different kinematic spaces (Movies 1–5, Fig. 5). The basigrade style is characterized by species with increased coxa linear velocities and attempted to capture prey that were further away (Fig. 4). Anterograde species tend to have high coxa starting angles (forelegs held roughly perpendicular to the pronotum), greater angular velocities, greater lateral displacement, and higher predator prey angles (loading negatively on kPC3). This style is employed by *H. coronatus* (coxa starting angle=87.0±5.8 deg mean±s.e.m.) and *P. wahlbergi* (coxa starting angle=61.2±4.5 deg), which are both Hymenopodid flower mimics in and corresponds with faster segment movements and lower predator prey distances (Table S2)*. P. chlorophaea* (coxa starting angle=47.6±4.2 deg) a Mantid stick mimic will also sometimes utilize anterograde strikes, but on average, is just shy of being considered an obligate anterograde hunter. Generalists, dead leaf mimics and *T. elegans* employed a basigrade hunting style, characterized by lower coxa starting angles (mean 29.1±1.2 deg, Table S2), lower predator prey angles, greater coxa linear velocity (faster coxa movement) and greater predator–prey distance.

The faster strikes of mantises employing the anterograde style may be related to the types of prey they capture. For example, Orchid mantises are known to specialize on insects that forage on inflorescences such as Diptera, Hymenoptera and Lepidoptera, using aggressive mimicry (Mizuno et al., 2014; O'Hanlon et al., 2014). This species was found to use anterograde strategies and have some of the fastest tibial and femoral angular velocities (Fig. S3 and Fig. 4). Such kinematics may allow for the capture of prey items that may only pass by in addition to those that land and would require prediction of the prey's flight path for success. *Pseudovates*, although considered a basigrade hunter by our strict demarcation, is the closest to anterograde in the sample; given the morphological convergence with the many eastern hemisphere Empusini, which prefer feeding on flying prey. *Pseudovates* and possibly the Vatini in general may represent an adaptive divergence from their fellow Mantidae and may be converging with the Empusidae on similar dietary habits as well (Reitze and Nentwig, 1991; Wieland and Svenson, 2018; L.G., pers. obs.). More research is warranted on the benefits of this hunting strategy in mantises, across the families and camouflage strategies.

The variation in prey capture attempts among mantises may be correlated to dietary differences, but information is lacking among this group (Oufiero, 2020; Prete et al., 2013b). Previous studies sometimes refer to mantises collectively as generalist sit and wait predators, attributing findings on a few taxa – typically species in the genera *Tenodera*, *Mantis* and *Hierodula –* to the order (e.g. Fagan et al., 2002; Fagan and Odell, 1996; McKimmie, 2000). Studies which investigate prey capture behavior and consumption across multiple groups suggest the opposite, however. In the ‘generalists’ *Mantis religiosa* and *Tenodera sinensis*, a low to moderate overlap in diet was found between the two introduced species despite their broad consumption across ecological guilds, suggesting potential microhabitat differences (Wilson Rankin et al., 2023). An examination of prey preference between six mantis species used five generalist taxa and one stick-mimicking species, *Empusa pennata*, and found that all species differed in the variety of prey accepted, with *E. pennata* having the narrowest preference (Reitze and Nentwig, 1991). Therefore, species mimicking and thus inhabiting different portions of the microhabitat may encounter different prey, resulting in correlated variation of prey capture morphology and function (Ferry and Higham, 2022). Variation in prey capture kinematics and subsequent convergence or divergence within clades may represent correlations to different types of prey, similar to other systems (Norton, 1991; Stinson and Deban, 2017). However, without detailed information on the ecology of mantis species, these relationships remain elusive (Prete et al., 2013a,b). Cursory research of predation strategy and our sampling of just a few species have highlighted variation in prey stimuli preference and overall prey capture kinematics, indicating that a catch-all label of Mantodea as generalist sit-and-wait predators is inaccurate.

Mantodea likely evolved from a cursorial ancestor and have diversified to mimic nearly every type of terrestrial foliage known including flowers, moss, lichen, leaves, grass, dead leaves, galls, detritus and sticks, as well as ants, wasps and rocks (Rivera and Svenson, 2016; Svenson and Whiting, 2009; Wieland and Svenson, 2018). While testing a small subset of these camouflage strategies, our results find that foreleg kinematics can converge between examined families and camouflage strategies despite differences in evolutionary history and linear morphology, an example of many-to-one mapping. The versatility of the raptorial forelegs is demonstrated by the kinematic components of each significant PC axis (Figs 3 and 4), where phylogenetic and camouflage groups vary in the foreleg velocities, amount of body used, where they attack prey, and the lateral displacement of the forelegs. The raptorial foreleg comprises three segments (Fig. 1C), each moving independently in both forelegs, resulting in many degrees of freedom during prey capture, which likely contributes to their versatility and ability to evolve vastly different camouflage strategies within related lineages. Similar kinematic results have been found among Amblypygi species, where the use of raptorial forelegs for prey capture varied among species, even among morphologically similar species (McLean et al., 2024).

The four camouflage strategies examined here (stick, flower, generalist and dead leaf) are anthropomorphically recognizable as such, but function differently within historical ecomorph groupings and between species, suggesting that in Mantodea, cryptic strategy and foreleg linear morphology are not consistent indicators of predation strategy. Thus, our understanding of the mantodean ecomorph needs re-evaluation, as we found that function and form are not correlated. *Theopropus elegans* for example*,* a diminutive Hymenopodid, is ostensibly a flower mimic; it has features associated with floral mimicry such as whitish coloration, leg lobes, raised vertex of the head and pronounced eyes with allometric affinity to other flower mimics (Fig. 2, Figs S3 and S4). However, it hunts with a basigrade style and converges kinematically with the generalist *S. limbata*, highlighting intrafamilial variation in hunting strategies. In contrast, the stick-mimicking Mantid *P. chlorophaea* convergently evolved an anterograde hunting strategy despite morphological affinity to the Deroplatyid *Euchomenella*, and phylogenetic proximity to generalist taxa (Figs 1 and 4). Although form (in terms of foreleg and metazona lengths) is dictated by phylogenetic proximity, function differs, as some mantises compensate kinematically to converge upon hunting strategies used in other taxa. A greater sampling of Mantodean diversity is required to test more proposed ecomorphs across greater taxonomic diversity, as our sampling represents ∼0.4% of known species (Brannoch et al., 2017; Svenson and Whiting, 2009). As a nearly cosmopolitan group, Mantodea offer a unique opportunity to examine the selection and diversification of predator camouflage ecomorphs at the order level across multiple lineages and continents. Future studies which incorporate diet are sorely needed to contextualize raptorial foreleg form and function diversity to understand the role these diverse predators play in their ecosystems. Furthermore, our examination of prey capture among camouflage strategies was done in a laboratory, common garden setting and may not reflect natural hunting behaviors when in the environments they are mimicking (Turko et al., 2023). Studies that examine predation strategies under more natural settings may reveal additional behavioral differences.

## Supplementary Material

10.1242/jexbio.250626_sup1 Supplementary information

Dataset 1. Raw data file of kinematic output for all trials used in R code (GarikipatiEA 2025.Rmd) for analyses and figures.

Dataset 2. R Markdown code that includes all data cleanup, transformation, analyses and raw figures.

Dataset 3. Html out generated by GarkipatiEA 2025.Rmd showing the data cleanup, transformation, analyses, and figures.
