# Supplementary material for: Many-to-one mapping in Mantodea: camouflage strategy and phylogeny drive strike variation in prey capture with raptorial forelegs
Source: J Exp Biol. 2025 Oct 13;228(19):jeb250626. doi: 10.1242/jeb.250626 (PMC12579948; doi:10.1242/jeb.250626)
Supplement: Supplementary information [file jexbio-228-250626-s1.pdf]

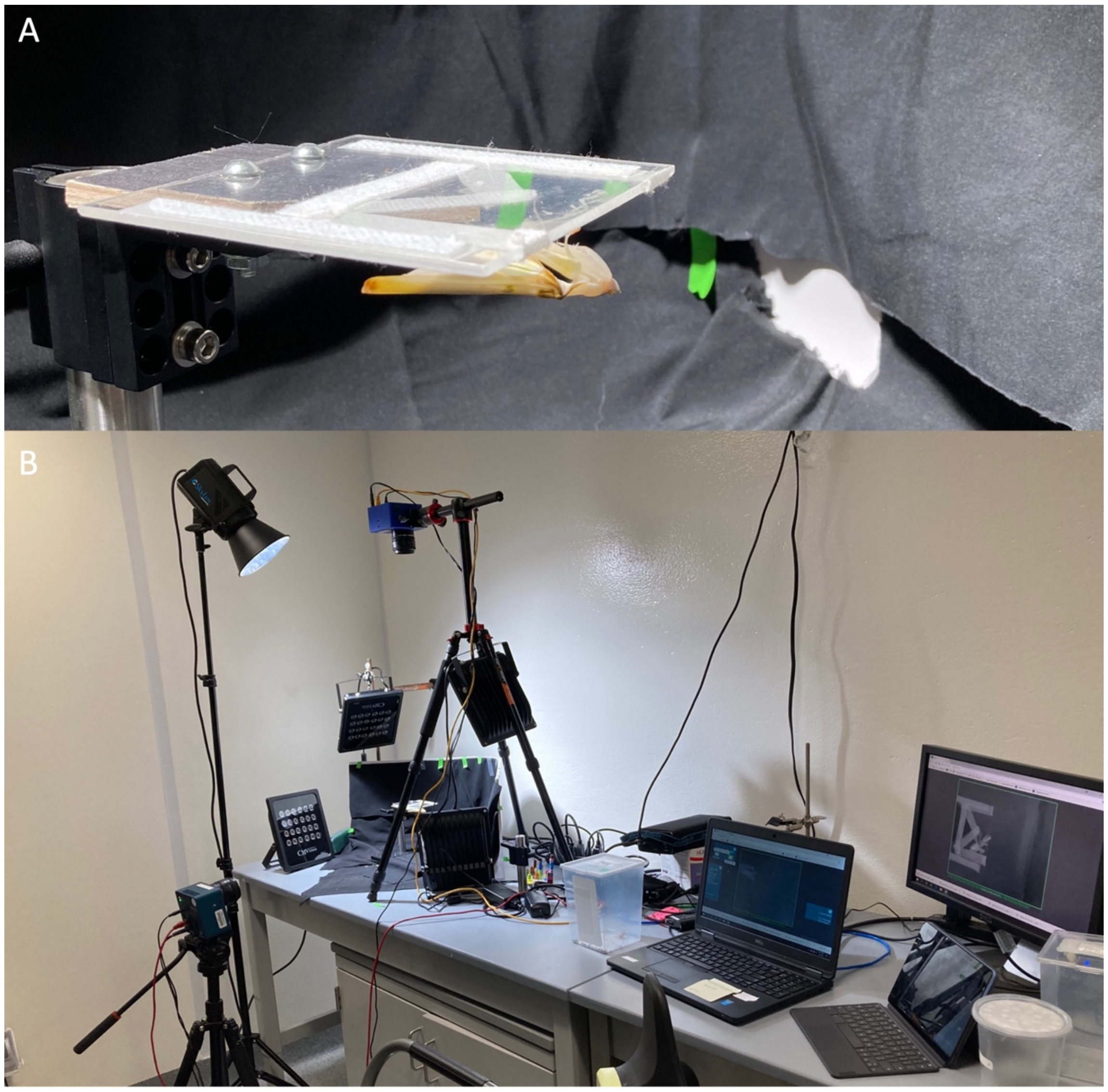

Fig. S1. Kinematic set up for praying mantis feeding trials A) *Hymenopus coronatus* on filming platform, awaiting trials. Lego calibrations taken in the space immediately in front of the platform. B) Two computers connected to different cameras to obtain dorsal and lateral videos during trials. The black background resolves to gray in the videos.

Journal of Experimental Biology • Supplementary information

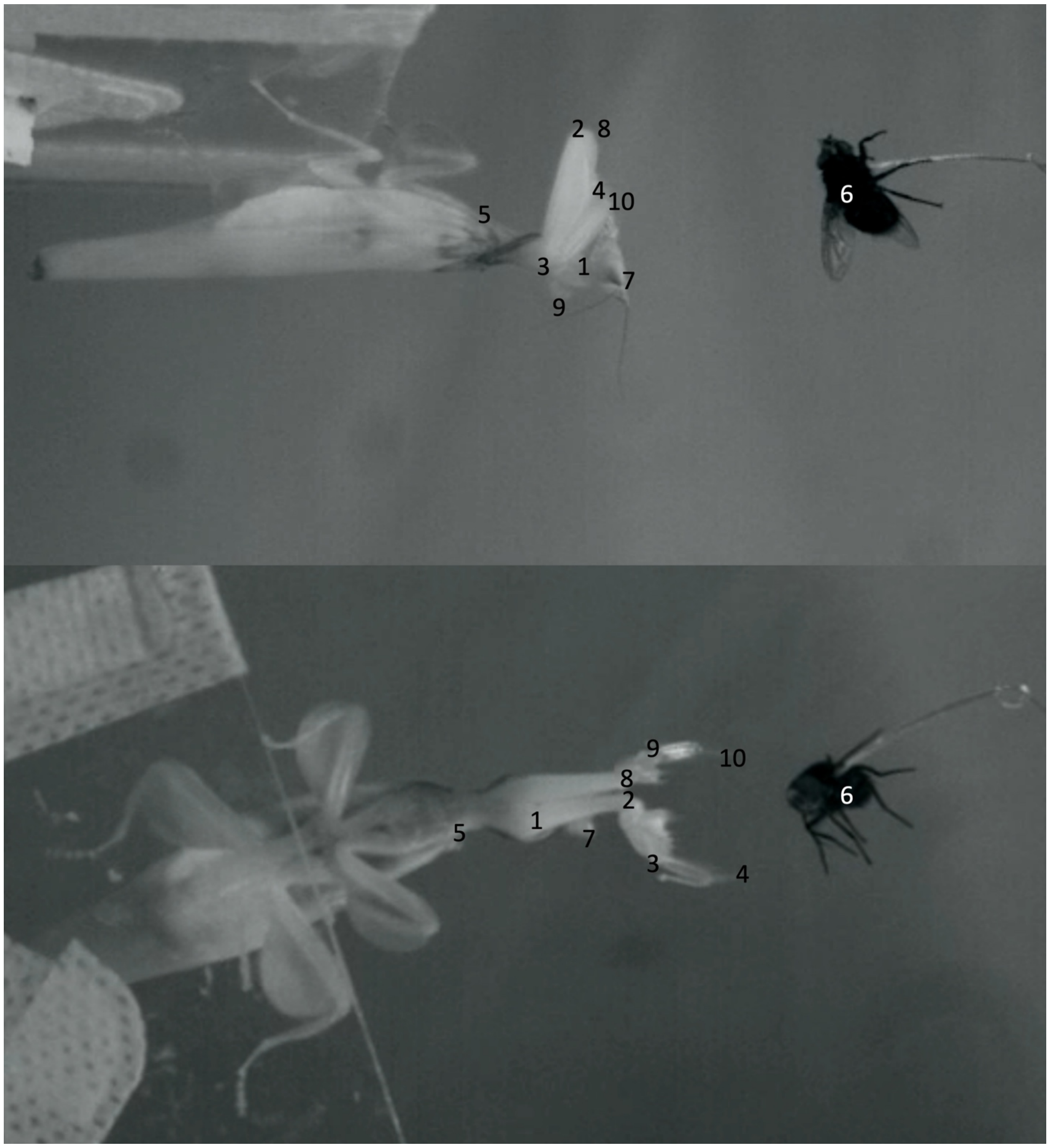

Fig. S2. Tracked points on the mantis. 1) Base of the right coxa; 2, 8) Ventral margin of the right and left trochanter respectively; 3, 9) Dorsal margin of the right and left forefemur respectively; 4, 10) Apex of the right and left foretibia respectively; 5) Base of the right mesocoxa; 6) Prey item

Journal of Experimental Biology • Supplementary information

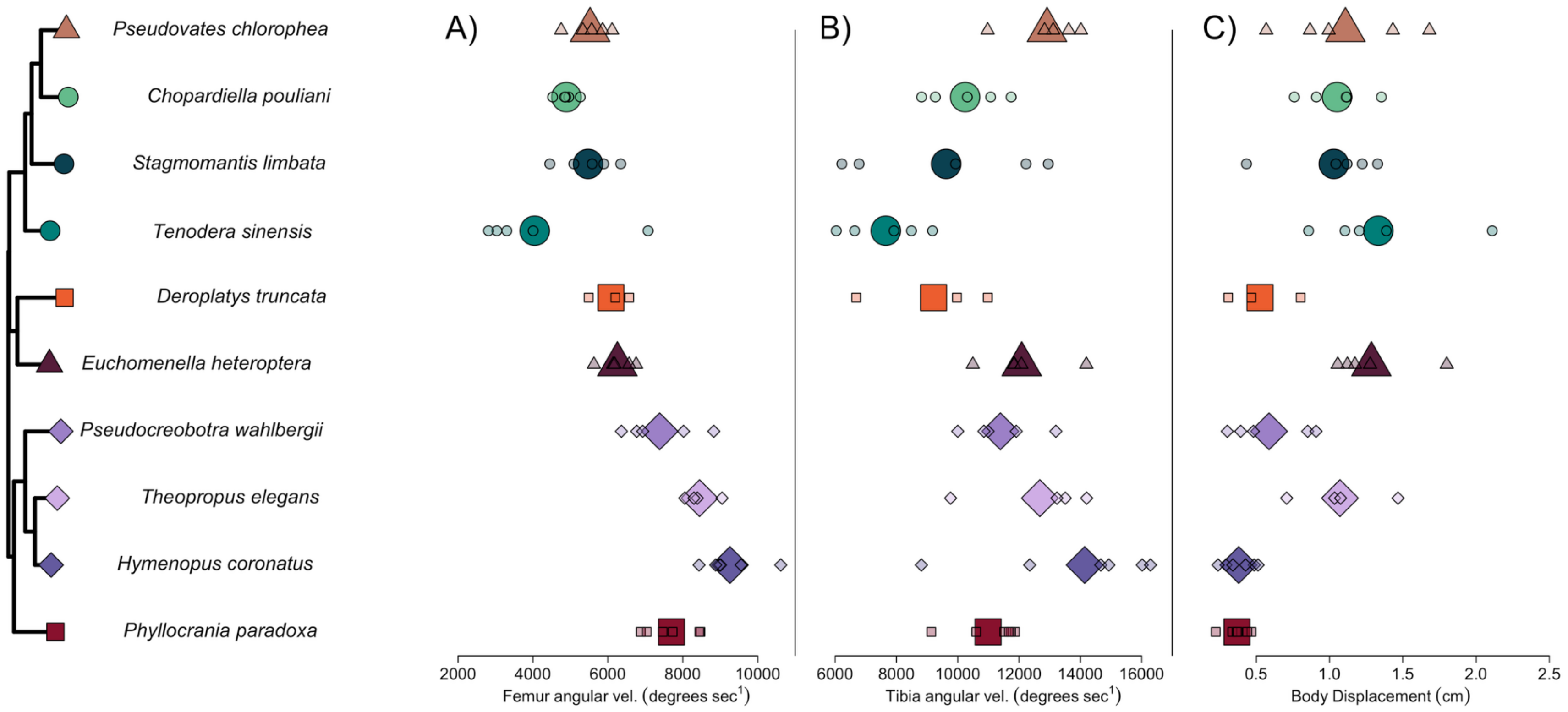

Fig. S3. Individual (smaller points) and species (larger points) means for some of the traits that have strong loadings on kinematic PC1. A) Femur angular velocity among species, B) tibia angular velocity, C) body displacement. Phylogeny from Fig. 1 provided as reference for species relationships. Colors and symbols match those in other figures. Circles (green) = generalists, square (organge/ red) = dead leaf, diamond (Purples) = flower, and triangle (browns) = sticks.

Journal of Experimental Biology • Supplementary information

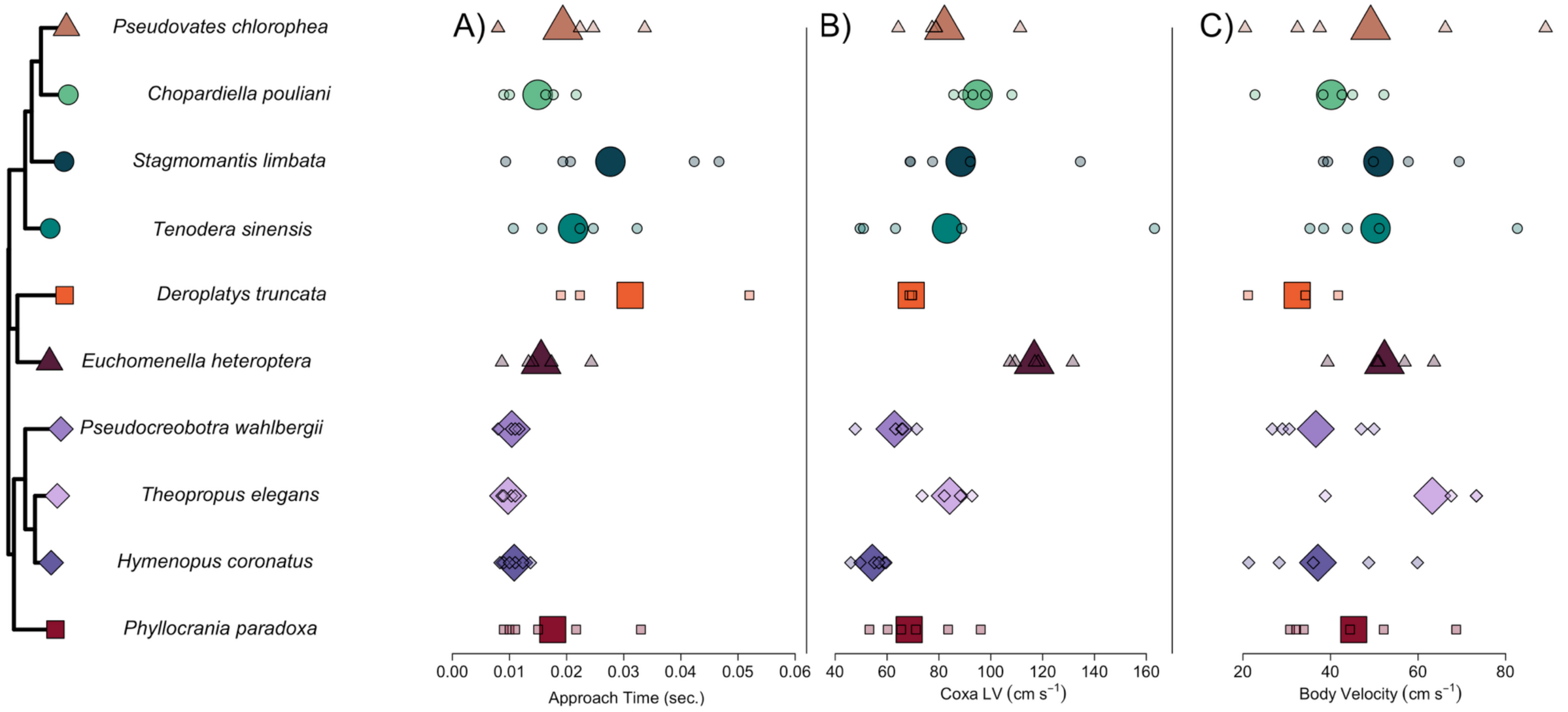

Fig. S4. Individual (smaller points) and species (larger points) means for some of the traits that have strong loadings on kinematic PC2. A) Approach time among species, B) Coxa linear velocity, C) body velocity. Phylogeny from Figure 1 provided as reference for species relationships. Colors and symbols match those in other figures. Circles (green) = generalists, square (orange/red) = dead leaf, diamond (Purples) = flower, and triangle (browns) = sticks.

Journal of Experimental Biology • Supplementary information

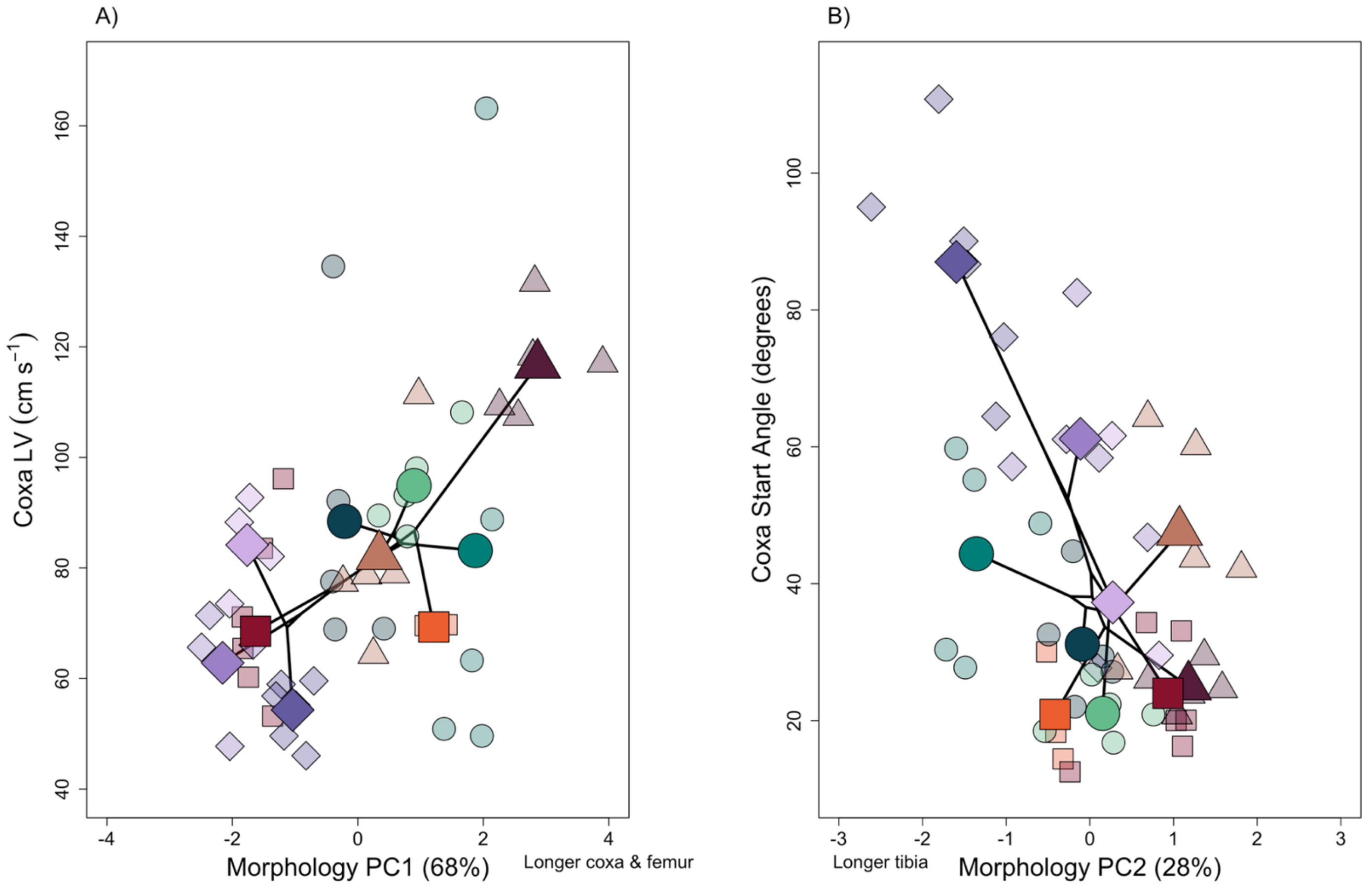

Fig. S5. Relationship between morphology PC 1 and coxa linear velocity (A) and morphology PC2 and coxa start angle (B). Smaller points are individual means; larger points are species means. Black lines are the phylogenetic relationships. Colors and symbols match those in other figures. Circles (green) = generalists, square (orange/red) = dead leaf, diamond (Purples) = flower, and triangle (browns) = sticks.

Table S1. Taxon Sampling and Categorization

Species	**Family: Subfamily**	**Ecomorph**
Hymenopus coronatus (Olivier, 1792) n=5	Hymenopodidae: Hymenopodinae	Flower
Theopropus elegans (Westwood, 1832) n=5	Hymenopodidae: Hymenopodinae	Flower
Pseudocreobtra wahlbergi (Stal, 1871) n=5	Hymenopodidae: Hymenopodinae	Flower
Phyllocrania paradoxa (Burmeister, 1838) n=5	Hymenopodidae: Phyllocraniinae	Dead Leaf
Deroplatys truncata (Guerin-Meneville, 1843) n=3	Deroplatyidae: Deroplatyinae	Dead Leaf
Euchomenella heteroptera (de Haan, 1842) n=5	Deroplatyidae: Deroplatyinae	Stick
Pseudovates chlorophaea Blanchard, 1836 n=5	Mantidae: Vatinae	Stick
Chopardiella pouliani Lombardo & Agabiti, 2001 n=5	Mantidae: Vatinae	Generalist
Stagmomantis limbata (Hahn, 1835) n=5	Mantidae: Stagmomantinae	Generalist
Tenodera sinensis (Saussure, 1871) n=5	Mantidae: Mantinae	Generalist

Journal of Experimental Biology • Supplementary information

Table S2. Mean trait values and standard errors for each of the 16 kinematic variables used in this study among the 10 species. Means represent the averages of the 3 fastest prey capture attempts from 3-5 females in each species. LV = linear velocity, AV = angular velocity, LD = lateral displacement, PP = predator prey, Disp = displacement, Dist = distance.

Means

Species	Coxa LV	Femur LV	Coxa AV	Femur AV	Tibia AV	Tibia LV	Coxa Start Angle	Approach Time	PP Angle	PP Dist	Body Vel	Body Disp	Sweep Time	Coxa/Femur LD	Femur/Tibia LD	Tibia LD
Chopardiella pouliani	97.3	127.6	3784.4	5074.0	10520.6	151.6	20.87	0.014	129.85	3.21	40.1	1.02	0.029	0.599	1.389	1.629
Deroplatys truncata	69.3	182.3	2271.0	6084.2	9211.8	149.5	20.91	0.031	108.89	2.66	32.4	0.53	0.021	0.694	1.553	1.450
Euchomenella heteroptera	116.7	185.8	3666.7	6253.3	12086.7	138.6	24.96	0.016	111.60	4.45	52.3	1.29	0.028	0.464	1.433	1.680
Hymenopus coronatus	54.3	190.6	3849.2	9258.7	14140.0	242.0	87.02	0.011	133.04	2.62	37.1	0.38	0.013	0.535	1.439	1.779
Phyllocrania paradoxa	68.5	148.5	3970.4	7697.3	10989.3	147.0	23.99	0.018	111.33	1.78	45.3	0.37	0.014	0.435	1.277	1.320
Pseudocreobotra wahlbergii	62.8	134.3	4667.3	7382.0	11390.1	164.1	61.18	0.010	127.31	2.10	36.6	0.59	0.015	0.654	1.645	1.818
Pseudovates chlorophea	82.1	128.2	3349.7	5524.2	12911.7	144.1	47.56	0.019	130.23	3.00	49.2	1.11	0.024	0.736	2.175	2.436
Stagmomantis limbata	88.4	123.5	3323.1	5470.5	9621.4	134.1	31.17	0.028	135.24	3.09	50.9	1.03	0.030	0.723	1.719	1.999
Tenodera sinensis	83.1	118.0	2555.6	4045.2	7650.5	137.1	44.38	0.021	142.71	3.56	50.3	1.33	0.037	0.824	1.907	1.899
Theopropus elegans	84.2	145.9	5863.3	8446.4	12678.7	145.7	37.28	0.010	136.54	2.90	63.3	1.07	0.019	0.579	1.520	1.958

Standard Errors

Species	Coxa LV	Femur LV	Coxa AV	Femur AV	Tibia AV	Tibia LV	Coxa Start Angle	Approach Time	PP Angle	PP Dist	Body Vel	Body Disp	Sweep Time	Coxa/Femur LD	Femur/Tibia LD	Tibia LD
Chopardiella pouliani	3.29	6.23	144.91	156.70	444.69	9.94	2.07	0.00	3.59	0.16	3.41	0.11	0.00	0.05	0.10	0.12
Deroplatys truncata	3.06	9.19	145.43	253.68	889.11	15.78	2.85	0.01	5.56	0.14	5.10	0.07	0.00	0.05	0.12	0.16
Euchomenella heteroptera	3.30	7.34	122.01	260.57	562.28	7.62	1.71	0.00	3.37	0.26	4.55	0.12	0.00	0.03	0.14	0.15

Journal of Experimental Biology • Supplementary information

Hymenopus coronatus	2.36	7.98	322.66	370.68	851.19	12.62	5.76	0.00	4.38	0.15	4.64	0.05	0.00	0.04	0.03	0.07
Phyllocrania paradoxa	4.23	8.85	255.13	392.50	597.80	10.91	3.00	0.00	4.49	0.12	6.59	0.05	0.00	0.02	0.07	0.10
Pseudocreobotra wahlbergii	3.98	9.94	253.11	435.41	566.01	11.17	4.54	0.00	3.05	0.18	4.06	0.08	0.00	0.05	0.07	0.10
Pseudovates chlorophea	5.74	6.05	194.71	165.65	463.65	9.86	4.15	0.01	4.59	0.15	7.61	0.17	0.00	0.07	0.14	0.16
Stagmomantis limbata	14.26	7.23	247.21	349.52	919.69	11.89	3.27	0.01	3.70	0.17	8.02	0.13	0.00	0.08	0.10	0.12
Tenodera sinensis	20.72	14.53	289.05	688.94	801.52	13.86	4.33	0.00	4.80	0.23	8.48	0.14	0.00	0.07	0.10	0.12
Theopropus elegans	4.37	5.46	251.26	296.27	603.15	9.83	4.56	0.00	4.06	0.18	6.11	0.11	0.00	0.03	0.05	0.08

Journal of Experimental Biology • Supplementary information

Table S3. Mean trait values and standard errors for each of the 4 morphological variables used in this study among the 10 species. Means represent the averages of the 3 fastest prey capture attempts from 3-5 females in each species.

Means

	Coxa length	Femur length	Tibia length	Metazona length
Chopardiella pouliani	1.5378	2.1751	0.8578	2.8658
Deroplatys truncata	1.6129	2.3676	0.9234	2.5814
Euchomenella heteroptera	1.9065	2.6675	0.7442	4.3694
Hymenopus coronatus	1.2744	1.6869	1.0517	0.8518
Phyllocrania paradoxa	1.0657	1.5062	0.7057	1.5518
Pseudocreobotra wahlbergii	1.0594	1.304	0.835	0.7949
Pseudovates chlorophea	1.4279	1.874	0.7423	3.1717
Stagmomantis limbata	1.3762	1.8953	0.8619	1.9414
Tenodera sinensis	1.8085	2.5208	1.0545	2.5297
Theopropus elegans	1.1178	1.4948	0.7751	0.9607

Standard Errors

Chopardiella pouliani	0.0268	0.0347	0.0257	0.0739
Deroplatys truncata	0.0408	0.0399	0.0164	0.0664
Euchomenella heteroptera	0.0227	0.0219	0.0202	0.0655
Hymenopus coronatus	0.0304	0.0129	0.0236	0.0408
Phyllocrania paradoxa	0.0146	0.0188	0.0229	0.0581
Pseudocreobotra wahlbergii	0.0277	0.0262	0.0161	0.0405
Pseudovates chlorophea	0.0288	0.0346	0.0203	0.07
Stagmomantis limbata	0.0277	0.0239	0.0179	0.052
Tenodera sinensis	0.0283	0.0442	0.0152	0.104

Journal of Experimental Biology • Supplementary information

Dataset 1. Raw data file of kinematic output for all trials used in R code (GarikipatiEA 2025.Rmd) for analyses and figures.

Available for download at
https://journals.biologists.com/jeb/article-lookup/doi/10.1242/jeb.250626#supplementary-data

Dataset 2. R Markdown code that includes all data cleanup, transformation, analyses and raw figures.

Available for download at
https://journals.biologists.com/jeb/article-lookup/doi/10.1242/jeb.250626#supplementary-data

Dataset 3. Html out generated by GarkipatiEA 2025.Rmd showing the data cleanup, transformation, analyses, and figures.

Available for download at
https://journals.biologists.com/jeb/article-lookup/doi/10.1242/jeb.250626#supplementary-data

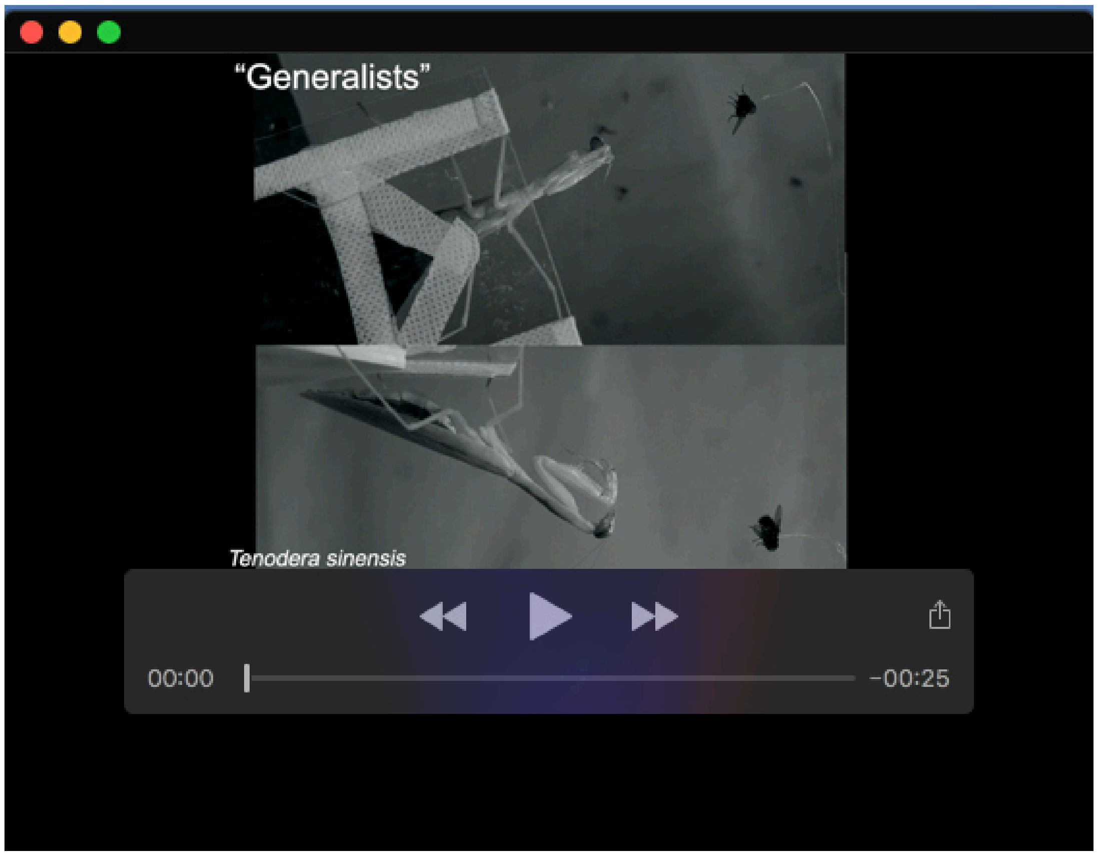

Movie 1. Representative videos of each of the species classified as generalists, which includes *Tenodera sinensis, Chopardiella pouliani,* and *Stagmomantis limbata*. All videos filmed at 1000 Hz, played back at 30Hz.

Journal of Experimental Biology • Supplementary information

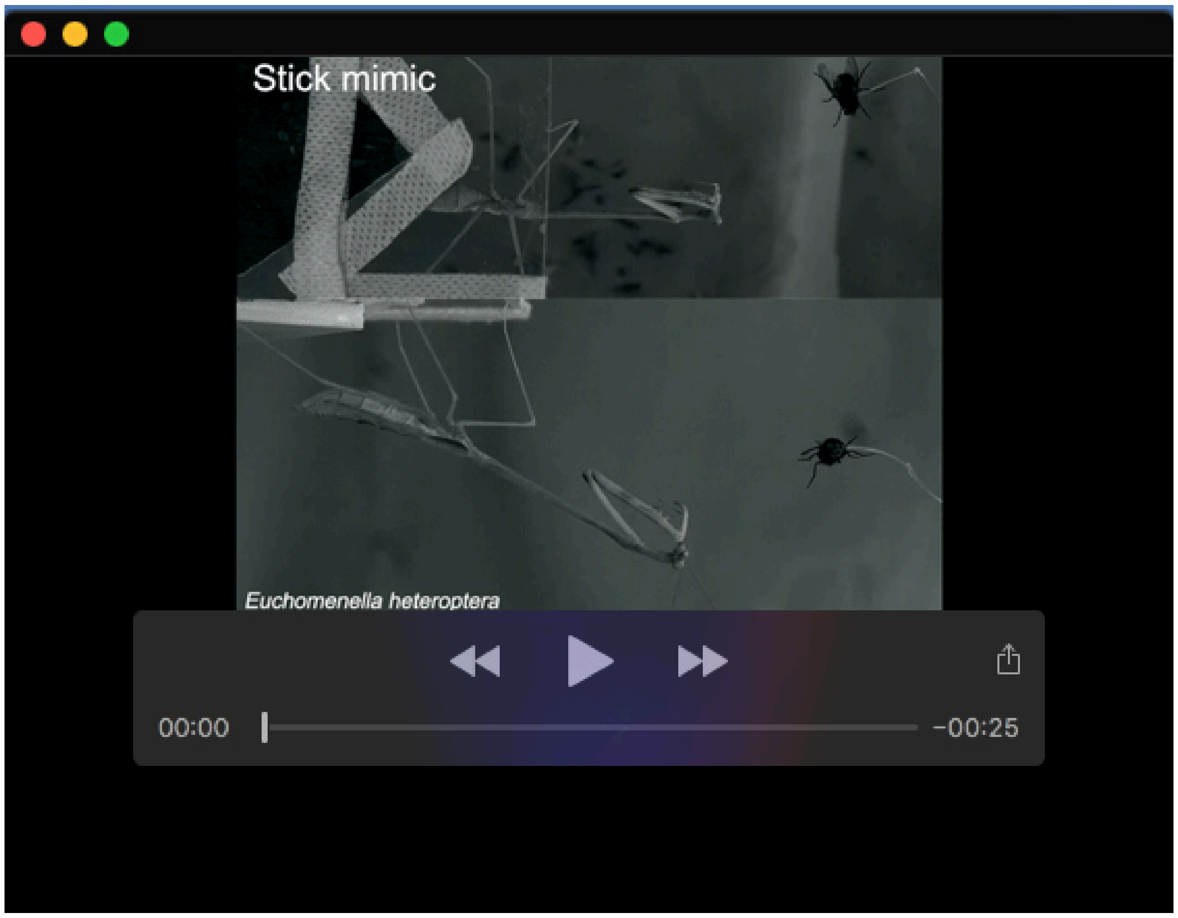

Movie 2. Representative videos of each of the species classified as stick mimics, which includes *Euchomenella heteroptera,* and *Pseudovates chlorophaea*. All videos filmed at 1000 Hz, played back at 30Hz.

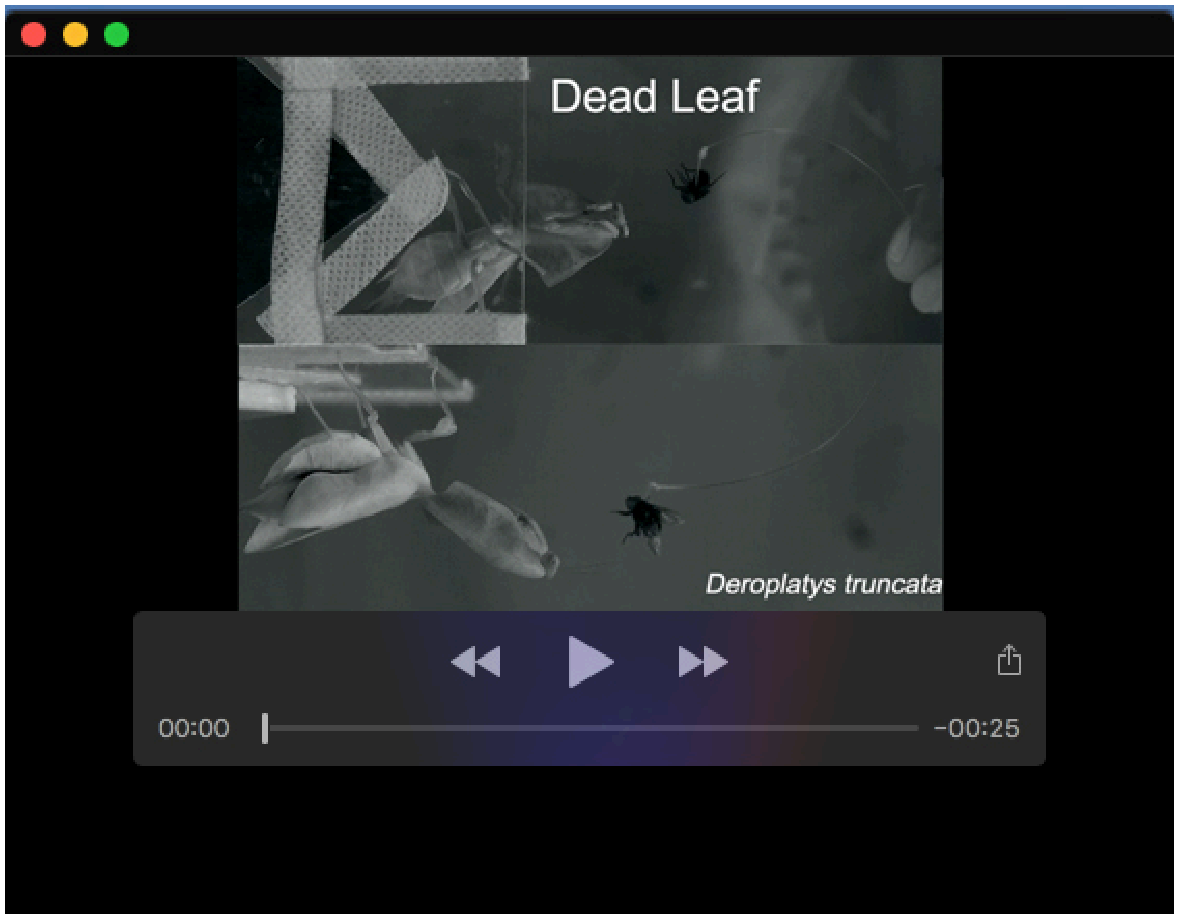

Movie 3. Representative videos of each of the species classified as stick mimics, which includes *Deroplatys truncata* and *Phyllocrania paradoxa*. All videos filmed at 1000 Hz, played back at 30Hz.

Journal of Experimental Biology • Supplementary information

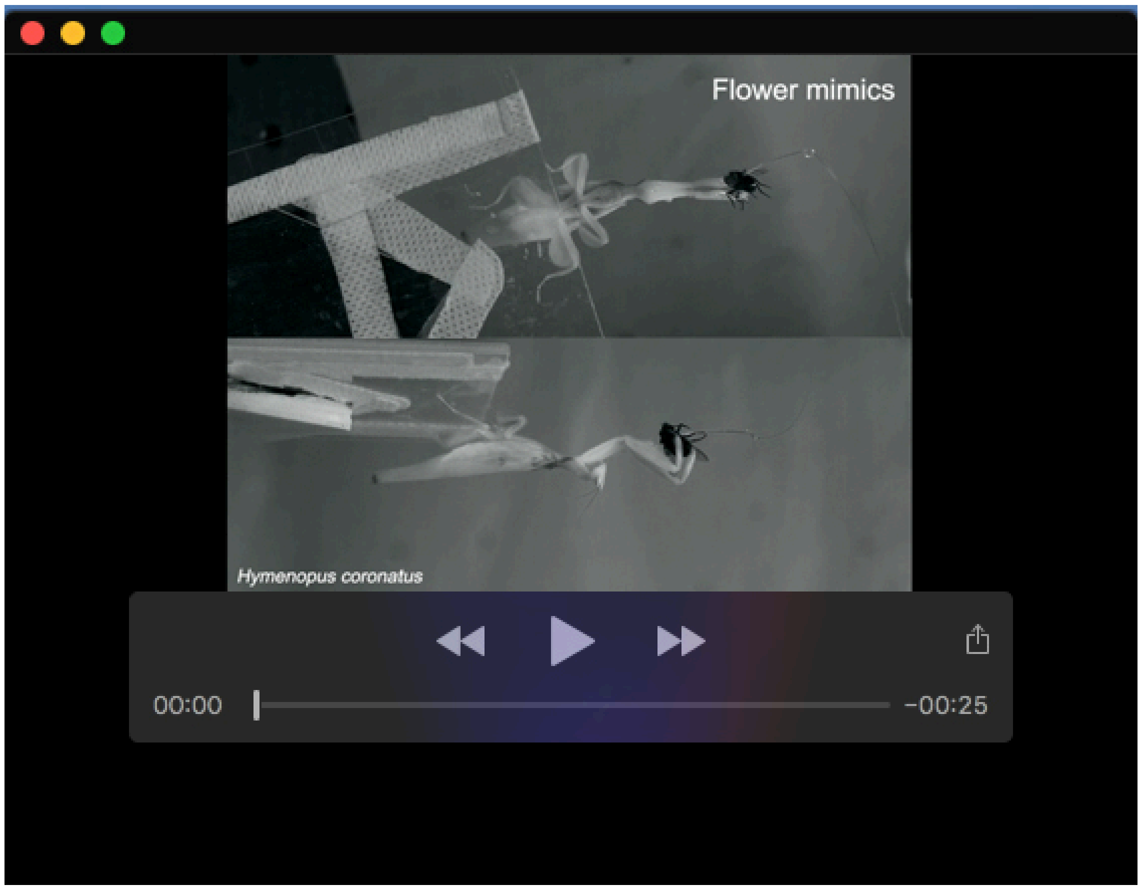

Movie 4. Representative videos of each of the species classified as flower mimics, which includes *Hymenopus coronatus, Pseudocreobotra wahlbergi,* and *Theopropus elegans*. All videos filmed at 1000 Hz, played back at 30Hz.

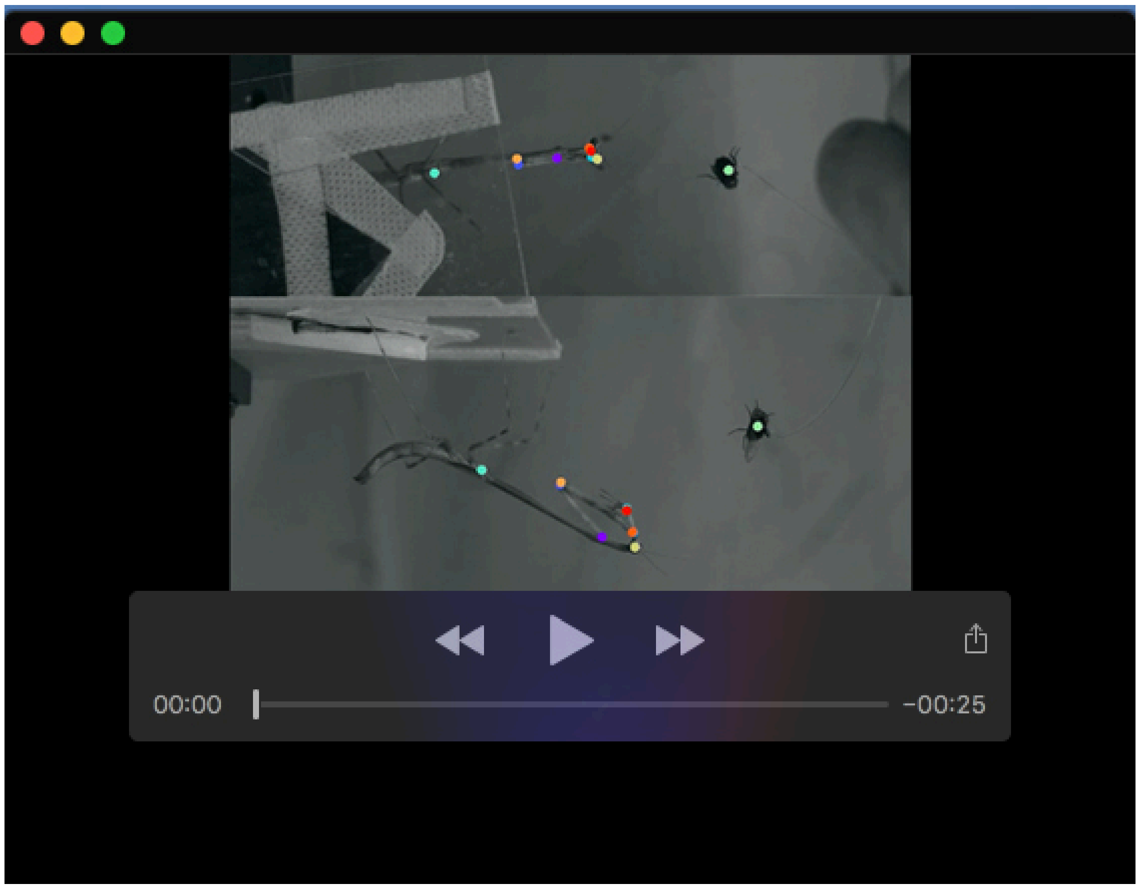

Movie 5. Example results of machine learning tracked points from DeepLabCut on *Euchomenella heteroptera*. These tracked points were manually cleaned up in DLTdv8.

Journal of Experimental Biology • Supplementary information
