# Supplementary material for: Many-to-one mapping in Mantodea: camouflage strategy and phylogeny drive strike variation in prey capture with raptorial forelegs
Source: J Exp Biol. 2025 Oct 13;228(19):jeb250626. doi: 10.1242/jeb.250626 (PMC12579948; doi:10.1242/jeb.250626)
Supplement: Dataset 3. Html out generated by GarkipatiEA 2025.Rmd showing the data cleanup, transformation, analyses, and figures. [file jexbio-228-250626-Dataset3.zip › GarikipatiEA-2025.html]

Garikipati EA 2025


# Garikipati EA 2025

#### CEO

#### 2025-07-16

# Overview

This R Markdown includes the code for the analyses and figures
associated with “Many to One Mapping in Mantodea: Camouflage Strategy
and Phylogeny Drive Strike Variation in Prey Capture with Raptorial
Forelegs”

To run the code, the following datasets are needed: “Mantis.nex.txt”
(from the Svenson and Whitting 2009 paper) “Lohit\_Final\_Log.csv”

## Read in libraries

```
library(phytools)
```

```
## Loading required package: ape
```

```
## Loading required package: maps
```

```
library(geiger)
library(MCMCglmm)
```

```
## Loading required package: Matrix
```

```
## Loading required package: coda
```

```
library(tidyverse)
```

```
## Warning: package 'ggplot2' was built under R version 4.3.3
```

```
## ── Attaching core tidyverse packages ──────────────────────── tidyverse 2.0.0 ──
## ✔ dplyr     1.1.3     ✔ readr     2.1.4
## ✔ forcats   1.0.0     ✔ stringr   1.5.0
## ✔ ggplot2   3.5.2     ✔ tibble    3.2.1
## ✔ lubridate 1.9.2     ✔ tidyr     1.3.0
## ✔ purrr     1.0.1
```

```
## ── Conflicts ────────────────────────────────────────── tidyverse_conflicts() ──
## ✖ tidyr::expand() masks Matrix::expand()
## ✖ dplyr::filter() masks stats::filter()
## ✖ dplyr::lag()    masks stats::lag()
## ✖ purrr::map()    masks maps::map()
## ✖ tidyr::pack()   masks Matrix::pack()
## ✖ tidyr::unpack() masks Matrix::unpack()
## ✖ dplyr::where()  masks ape::where()
## ℹ Use the conflicted package (<http://conflicted.r-lib.org/>) to force all conflicts to become errors
```

```
library(caper)
```

```
## Loading required package: MASS
## 
## Attaching package: 'MASS'
## 
## The following object is masked from 'package:dplyr':
## 
##     select
## 
## Loading required package: mvtnorm
```

```
library(emmeans)
library(MuMIn)
```

```
## Registered S3 method overwritten by 'MuMIn':
##   method    from 
##   nobs.pgls caper
```

```
library(vegan)
```

```
## Loading required package: permute
## Loading required package: lattice
## This is vegan 2.6-4
## 
## Attaching package: 'vegan'
## 
## The following object is masked from 'package:phytools':
## 
##     scores
```

## Read in data files and transform variables

```
tree<-read.nexus("Mantis.nex.txt")
data<-read.csv("Lohit_Final_Log.csv")
#take absolute values for tibia since it is a flexion resulting in a negative value
data$abs_tibia_AV <-abs(data$Tibia_AV_min)
data$abs_tibia_LV <-abs(data$Tibia_LV_min)
data<-data[complete.cases(data[,16:95]), ]


#trim rows for ecomorph and species, remove blank spaces
data$Species <- trimws(data$Species) #trimws removes leading or trailing character space
test_sp_df <- as.factor(data$Species)
droplevels(test_sp_df, exclude = "")
```

```
##   [1] Hymenopus coronatus        Hymenopus coronatus       
##   [3] Hymenopus coronatus        Hymenopus coronatus       
##   [5] Hymenopus coronatus        Hymenopus coronatus       
##   [7] Hymenopus coronatus        Hymenopus coronatus       
##   [9] Hymenopus coronatus        Hymenopus coronatus       
##  [11] Hymenopus coronatus        Hymenopus coronatus       
##  [13] Hymenopus coronatus        Hymenopus coronatus       
##  [15] Hymenopus coronatus        Hymenopus coronatus       
##  [17] Hymenopus coronatus        Hymenopus coronatus       
##  [19] Hymenopus coronatus        Hymenopus coronatus       
##  [21] Hymenopus coronatus        Hymenopus coronatus       
##  [23] Hymenopus coronatus        Tenodera sinensis         
##  [25] Tenodera sinensis          Tenodera sinensis         
##  [27] Tenodera sinensis          Tenodera sinensis         
##  [29] Tenodera sinensis          Tenodera sinensis         
##  [31] Tenodera sinensis          Tenodera sinensis         
##  [33] Tenodera sinensis          Tenodera sinensis         
##  [35] Tenodera sinensis          Tenodera sinensis         
##  [37] Tenodera sinensis          Tenodera sinensis         
##  [39] Tenodera sinensis          Tenodera sinensis         
##  [41] Tenodera sinensis          Tenodera sinensis         
##  [43] Tenodera sinensis          Tenodera sinensis         
##  [45] Tenodera sinensis          Tenodera sinensis         
##  [47] Tenodera sinensis          Tenodera sinensis         
##  [49] Stagmomantis limbata       Stagmomantis limbata      
##  [51] Stagmomantis limbata       Stagmomantis limbata      
##  [53] Stagmomantis limbata       Stagmomantis limbata      
##  [55] Stagmomantis limbata       Stagmomantis limbata      
##  [57] Stagmomantis limbata       Stagmomantis limbata      
##  [59] Stagmomantis limbata       Stagmomantis limbata      
##  [61] Stagmomantis limbata       Stagmomantis limbata      
##  [63] Stagmomantis limbata       Stagmomantis limbata      
##  [65] Stagmomantis limbata       Stagmomantis limbata      
##  [67] Stagmomantis limbata       Stagmomantis limbata      
##  [69] Stagmomantis limbata       Stagmomantis limbata      
##  [71] Stagmomantis limbata       Stagmomantis limbata      
##  [73] Chopardiella pouliani      Chopardiella pouliani     
##  [75] Chopardiella pouliani      Chopardiella pouliani     
##  [77] Chopardiella pouliani      Chopardiella pouliani     
##  [79] Chopardiella pouliani      Chopardiella pouliani     
##  [81] Chopardiella pouliani      Chopardiella pouliani     
##  [83] Chopardiella pouliani      Chopardiella pouliani     
##  [85] Chopardiella pouliani      Chopardiella pouliani     
##  [87] Chopardiella pouliani      Chopardiella pouliani     
##  [89] Chopardiella pouliani      Chopardiella pouliani     
##  [91] Chopardiella pouliani      Chopardiella pouliani     
##  [93] Chopardiella pouliani      Chopardiella pouliani     
##  [95] Chopardiella pouliani      Chopardiella pouliani     
##  [97] Chopardiella pouliani      Chopardiella pouliani     
##  [99] Chopardiella pouliani      Chopardiella pouliani     
## [101] Pseudovates chlorophea     Pseudovates chlorophea    
## [103] Pseudovates chlorophea     Pseudovates chlorophea    
## [105] Pseudovates chlorophea     Pseudovates chlorophea    
## [107] Pseudovates chlorophea     Pseudovates chlorophea    
## [109] Pseudovates chlorophea     Pseudovates chlorophea    
## [111] Pseudovates chlorophea     Pseudovates chlorophea    
## [113] Pseudovates chlorophea     Pseudovates chlorophea    
## [115] Pseudovates chlorophea     Pseudovates chlorophea    
## [117] Pseudovates chlorophea     Pseudovates chlorophea    
## [119] Pseudovates chlorophea     Pseudovates chlorophea    
## [121] Pseudovates chlorophea     Pseudovates chlorophea    
## [123] Pseudovates chlorophea     Pseudovates chlorophea    
## [125] Pseudovates chlorophea     Theopropus elegans        
## [127] Theopropus elegans         Theopropus elegans        
## [129] Theopropus elegans         Theopropus elegans        
## [131] Theopropus elegans         Theopropus elegans        
## [133] Theopropus elegans         Theopropus elegans        
## [135] Theopropus elegans         Theopropus elegans        
## [137] Theopropus elegans         Theopropus elegans        
## [139] Theopropus elegans         Theopropus elegans        
## [141] Theopropus elegans         Theopropus elegans        
## [143] Theopropus elegans         Euchomenella heteroptera  
## [145] Euchomenella heteroptera   Euchomenella heteroptera  
## [147] Euchomenella heteroptera   Euchomenella heteroptera  
## [149] Euchomenella heteroptera   Euchomenella heteroptera  
## [151] Euchomenella heteroptera   Euchomenella heteroptera  
## [153] Euchomenella heteroptera   Euchomenella heteroptera  
## [155] Euchomenella heteroptera   Euchomenella heteroptera  
## [157] Euchomenella heteroptera   Euchomenella heteroptera  
## [159] Euchomenella heteroptera   Euchomenella heteroptera  
## [161] Euchomenella heteroptera   Euchomenella heteroptera  
## [163] Euchomenella heteroptera   Euchomenella heteroptera  
## [165] Euchomenella heteroptera   Euchomenella heteroptera  
## [167] Euchomenella heteroptera   Euchomenella heteroptera  
## [169] Pseudocreobotra wahlbergii Pseudocreobotra wahlbergii
## [171] Pseudocreobotra wahlbergii Pseudocreobotra wahlbergii
## [173] Pseudocreobotra wahlbergii Pseudocreobotra wahlbergii
## [175] Pseudocreobotra wahlbergii Pseudocreobotra wahlbergii
## [177] Pseudocreobotra wahlbergii Pseudocreobotra wahlbergii
## [179] Pseudocreobotra wahlbergii Pseudocreobotra wahlbergii
## [181] Pseudocreobotra wahlbergii Pseudocreobotra wahlbergii
## [183] Pseudocreobotra wahlbergii Pseudocreobotra wahlbergii
## [185] Pseudocreobotra wahlbergii Pseudocreobotra wahlbergii
## [187] Pseudocreobotra wahlbergii Pseudocreobotra wahlbergii
## [189] Pseudocreobotra wahlbergii Phyllocrania paradoxa     
## [191] Phyllocrania paradoxa      Phyllocrania paradoxa     
## [193] Phyllocrania paradoxa      Phyllocrania paradoxa     
## [195] Phyllocrania paradoxa      Phyllocrania paradoxa     
## [197] Phyllocrania paradoxa      Phyllocrania paradoxa     
## [199] Phyllocrania paradoxa      Phyllocrania paradoxa     
## [201] Phyllocrania paradoxa      Phyllocrania paradoxa     
## [203] Phyllocrania paradoxa      Phyllocrania paradoxa     
## [205] Phyllocrania paradoxa      Phyllocrania paradoxa     
## [207] Phyllocrania paradoxa      Phyllocrania paradoxa     
## [209] Phyllocrania paradoxa      Phyllocrania paradoxa     
## [211] Phyllocrania paradoxa      Phyllocrania paradoxa     
## [213] Phyllocrania paradoxa      Phyllocrania paradoxa     
## [215] Deroplatys truncata        Deroplatys truncata       
## [217] Deroplatys truncata        Deroplatys truncata       
## [219] Deroplatys truncata        Deroplatys truncata       
## [221] Deroplatys truncata        Deroplatys truncata       
## [223] Deroplatys truncata        Deroplatys truncata       
## [225] Deroplatys truncata        Deroplatys truncata       
## 10 Levels: Chopardiella pouliani ... Theopropus elegans
```

```
data$Species <- test_sp_df

data$Ecomorph <- trimws(data$Ecomorph) #trimws removes leading or trailing character space
test_eco_df <- as.factor(data$Ecomorph)
droplevels(test_eco_df, exclude = "")
```

```
##   [1] Flower     Flower     Flower     Flower     Flower     Flower    
##   [7] Flower     Flower     Flower     Flower     Flower     Flower    
##  [13] Flower     Flower     Flower     Flower     Flower     Flower    
##  [19] Flower     Flower     Flower     Flower     Flower     Generalist
##  [25] Generalist Generalist Generalist Generalist Generalist Generalist
##  [31] Generalist Generalist Generalist Generalist Generalist Generalist
##  [37] Generalist Generalist Generalist Generalist Generalist Generalist
##  [43] Generalist Generalist Generalist Generalist Generalist Generalist
##  [49] Generalist Generalist Generalist Generalist Generalist Generalist
##  [55] Generalist Generalist Generalist Generalist Generalist Generalist
##  [61] Generalist Generalist Generalist Generalist Generalist Generalist
##  [67] Generalist Generalist Generalist Generalist Generalist Generalist
##  [73] Generalist Generalist Generalist Generalist Generalist Generalist
##  [79] Generalist Generalist Generalist Generalist Generalist Generalist
##  [85] Generalist Generalist Generalist Generalist Generalist Generalist
##  [91] Generalist Generalist Generalist Generalist Generalist Generalist
##  [97] Generalist Generalist Generalist Generalist Stick      Stick     
## [103] Stick      Stick      Stick      Stick      Stick      Stick     
## [109] Stick      Stick      Stick      Stick      Stick      Stick     
## [115] Stick      Stick      Stick      Stick      Stick      Stick     
## [121] Stick      Stick      Stick      Stick      Stick      Flower    
## [127] Flower     Flower     Flower     Flower     Flower     Flower    
## [133] Flower     Flower     Flower     Flower     Flower     Flower    
## [139] Flower     Flower     Flower     Flower     Flower     Stick     
## [145] Stick      Stick      Stick      Stick      Stick      Stick     
## [151] Stick      Stick      Stick      Stick      Stick      Stick     
## [157] Stick      Stick      Stick      Stick      Stick      Stick     
## [163] Stick      Stick      Stick      Stick      Stick      Stick     
## [169] Flower     Flower     Flower     Flower     Flower     Flower    
## [175] Flower     Flower     Flower     Flower     Flower     Flower    
## [181] Flower     Flower     Flower     Flower     Flower     Flower    
## [187] Flower     Flower     Flower     Dead Leaf  Dead Leaf  Dead Leaf 
## [193] Dead Leaf  Dead Leaf  Dead Leaf  Dead Leaf  Dead Leaf  Dead Leaf 
## [199] Dead Leaf  Dead Leaf  Dead Leaf  Dead Leaf  Dead Leaf  Dead Leaf 
## [205] Dead Leaf  Dead Leaf  Dead Leaf  Dead Leaf  Dead Leaf  Dead Leaf 
## [211] Dead Leaf  Dead Leaf  Dead Leaf  Dead Leaf  Dead Leaf  Dead Leaf 
## [217] Dead Leaf  Dead Leaf  Dead Leaf  Dead Leaf  Dead Leaf  Dead Leaf 
## [223] Dead Leaf  Dead Leaf  Dead Leaf  Dead Leaf 
## Levels: Dead Leaf Flower Generalist Stick
```

```
data$Ecomorph <- test_eco_df

#get three fastest strikes based upon tibia and femur angular velocities
max.data <- data%>% group_by(Mantis) %>% arrange(desc(abs_tibia_AV), desc(Femur_AV)) %>%
  slice(1:3) %>%
  ungroup()
 max.data<-as.data.frame(max.data) 

#aggregate by species 
data.1<-aggregate(max.data[,16:93], by=list(Tip.label=max.data$Phylo,max.data$Species),
FUN=mean, na.rm=TRUE)
#tree<-read.nexus("Mantis.nex.txt")
colnames(data.1)[2] ="species"
#prunning the tree to match morphology data
row.names(data.1)<-data.1[,1]
foo<-name.check(tree, data.1)
print(foo)
```

```
## $tree_not_data
##   [1] "Acanthops_falcataria_MN112"          
##   [2] "Acanthops_sp._MN085"                 
##   [3] "Acontista_sp._MN111"                 
##   [4] "Acromantis_insularis_MN075"          
##   [5] "Acromantis_montana_MN082"            
##   [6] "Acromantis_sp._MN004"                
##   [7] "Acromantis_sp._MN321"                
##   [8] "Acromantis_sp._MN339"                
##   [9] "Aethalochroa_sp._MN264"              
##  [10] "Amantis_biroi_MN083"                 
##  [11] "Amantis_reticulata_MN003"            
##  [12] "Amantis_sp._MN209"                   
##  [13] "Amantis_tristis_MN333"               
##  [14] "Amorphoscelis_annulicornis_MN061"    
##  [15] "Amorphoscelis_austrogermanica_MN176" 
##  [16] "Amorphoscelis_borneana_MN103"        
##  [17] "Amorphoscelis_borneana_MN318"        
##  [18] "Amorphoscelis_singaporana_MN081"     
##  [19] "Amorphoscelis_sp._MN240"             
##  [20] "Amorphoscelis_sp._MN317"             
##  [21] "Amorphoscelis_sp._MN327"             
##  [22] "Amorphoscelis_sp._MN337"             
##  [23] "Anasigerpes_bifasciata_MN241"        
##  [24] "Anaxarcha_intermedia_MN253"          
##  [25] "Anaxarcha_limbata_MN080"             
##  [26] "Antemna_rapax_MN147"                 
##  [27] "Antistia_maculipennis_MN305"         
##  [28] "Archimantis_sobrina_MN012"           
##  [29] "Austrovates_variegata_MN084"         
##  [30] "Bantia_werneri_MN115"                
##  [31] "Bimantis_malaccana_MN331"            
##  [32] "Blatta_orientalis_BL097"             
##  [33] "Blattella_germanica_BL082"           
##  [34] "Blepharopsis_mendica_MN270"          
##  [35] "Bolbe_pallida_MN021"                 
##  [36] "Bolbe_pygmea_MN040"                  
##  [37] "Bolbella_punctigera_MN189"           
##  [38] "Bolbena_hottentotta_MN051"           
##  [39] "Bolbena_hottentotta_MN258"           
##  [40] "Bolbena_maraisi_MN043"               
##  [41] "Bolbena_sp._MN292"                   
##  [42] "Brunneria_sp._MN124"                 
##  [43] "Calofulcinia_sp._MN251"              
##  [44] "Camelomantis_moultoni_MN130"         
##  [45] "Cardioptera_squalodon_MN178"         
##  [46] "Carrikerella_ceratophora_MN148"      
##  [47] "Caudatoscelis_marmorata_MN239"       
##  [48] "Ceratocrania_macra_MN328"            
##  [49] "Ceratomantis_ghatei_MN204"           
##  [50] "Ceratomantis_kimberlae_MN326"        
##  [51] "Chaeteessa_valida_MN217"             
##  [52] "Chloroharpax_modesta_MN234"          
##  [53] "Choeradodis_rhombicollis_MN016"      
##  [54] "Choeradodis_stalii_MN127"            
##  [55] "Chroicoptera_saussurei_MN182"        
##  [56] "Chrysomantis_cachani_MN225"          
##  [57] "Chrysomantis_sp._MN005"              
##  [58] "Cilnia_humeralis_MN308"              
##  [59] "Citharomantis_falcata_MN102"         
##  [60] "Ciulfina_biseriata_MN036"            
##  [61] "Cliomantis_cornuta_MN055"            
##  [62] "Cliomantis_obscura_MN054"            
##  [63] "Compsothespis_sp._MN282"             
##  [64] "Congoharpax_aberrans_MN229"          
##  [65] "Coptopteryx_sp._MN059"               
##  [66] "Coptopteryx_sp._MN136"               
##  [67] "Coptotermes_lacteus_IS041"           
##  [68] "Creobroter_apicalis_MN072"           
##  [69] "Creobroter_laevicollis_MN073"        
##  [70] "Creobroter_sp._MN092"                
##  [71] "Cryptocercus_kyebangensis_BL115"     
##  [72] "Cryptocercus_russie_BL119"           
##  [73] "Cryptocercus_scioto_BL120"           
##  [74] "Cryptotermes_brevis_IS021"           
##  [75] "Dactylopteryx_flexuosa_MN242"        
##  [76] "Danuria_thunbergi_MN277"             
##  [77] "Danuriini_sp._MN032"                 
##  [78] "Danuriini_sp._MN307"                 
##  [79] "Deiphobe_sp._MN256"                  
##  [80] "Deiphobe_sp._MN265"                  
##  [81] "Deiphobella_laticeps_MN066"          
##  [82] "Deroplatys_desiccata_MN348"          
##  [83] "Deroplatys_rhombica_MN272"           
##  [84] "Deroplatys_rhombica_MN325"           
##  [85] "Deroplatys_rhombica_MN347"           
##  [86] "Deroplatys_sp._MN168"                
##  [87] "Didymocorypha_lanceolata_MN087"      
##  [88] "Dysaules_himalayanus_MN088"          
##  [89] "Dysaules_longicollis_MN202"          
##  [90] "Dysaules_sp._MN067"                  
##  [91] "Dystacta_alticeps_MN187"             
##  [92] "Dystacta_alticeps_MN188"             
##  [93] "Dystactula_grisea_MN197"             
##  [94] "Elmantis_nira_MN254"                 
##  [95] "Elmantis_nira_MN296"                 
##  [96] "Elmantis_trincomaliae_MN071"         
##  [97] "Elmantis_trincomaliae_MN261"         
##  [98] "Empusa_guttula_MN132"                
##  [99] "Empusa_sp._MN131"                    
## [100] "Entella_delalandi_MN183"             
## [101] "Entella_orientalis_MN304"            
## [102] "Entelloptera_rogenhoferi_MN300"      
## [103] "Eomantis_guttatipennis_MN074"        
## [104] "Eomantis_iridipennis_MN208"          
## [105] "Ephestiasula_intermedia_MN063"       
## [106] "Ephippiomantis_ophirensis_MN330"     
## [107] "Episcopomantis_chalybea_MN034"       
## [108] "Eremiaphila_rotundipennis_MN064"     
## [109] "Eremiaphila_sp._MN192"               
## [110] "Eremoplana_iufelix_MN159"            
## [111] "Euantissa_pulchra_MN077"             
## [112] "Euantissa_pulchra_MN201"             
## [113] "Eumusonia_livida_MN123"              
## [114] "Galepsus_ulricae_MN259"              
## [115] "Galinthias_amoena_MN266"             
## [116] "Galinthias_amoena_MN288"             
## [117] "Gildella_suavis_MN098"               
## [118] "Gimantis_insularis_MN100"            
## [119] "Gimantis_insularis_MN332"            
## [120] "Gongylus_gongylodes_MN006"           
## [121] "Gonypeta_borneana_MN097"             
## [122] "Gonypeta_borneana_MN324"             
## [123] "Gonypetella_sp._MN293"               
## [124] "Gonypetyllis_semuncialis_MN089"      
## [125] "Gonypetyllis_semuncialis_MN210"      
## [126] "Gyna_capucina_BL048"                 
## [127] "Gyromantis_occidentalis_MN053"       
## [128] "Gyromantis_occidentalis_MN056"       
## [129] "Haania_lobiceps_MN315"               
## [130] "Hagiomantis_superba_MN215"           
## [131] "Hapalomantis_congica_katangica_MN291"
## [132] "Hapalomantis_orba_MN280"             
## [133] "Hapalopeza_nilgirica_MN070"          
## [134] "Hapalopeza_nitens_MN316"             
## [135] "Hestiasula_inermis_MN199"            
## [136] "Hestiasula_masoni_MN297"             
## [137] "Hestiasula_phyllopus_MN340"          
## [138] "Heterochaeta_strachani_MN160"        
## [139] "Heterochaetula_fissispinis_MN207"    
## [140] "Hierodula_schultzei_MN044"           
## [141] "Hierodula_sp._MN018"                 
## [142] "Hierodula_sp._MN019"                 
## [143] "Hierodula_sp._MN343"                 
## [144] "Hierodulella_celebensis_MN345"       
## [145] "Hierodulella_reticulata_MN342"       
## [146] "Hodotermes_mossambicus_IS014"        
## [147] "Hoplocorypha_sp._MN035"              
## [148] "Hoplocorypha_sp._MN042"              
## [149] "Hoplocorypha_sp._MN139"              
## [150] "Hoplocorypha_sp._MN190"              
## [151] "Humbertiella_ocularis_MN093"         
## [152] "Humbertiella_ocularis_MN334"         
## [153] "Humbertiella_similis_MN069"          
## [154] "Idolomantis_diabolica_MN193"         
## [155] "Idolomorpha_dentifrons_MN175"        
## [156] "Indomenella_indica_MN079"            
## [157] "Iris_oratoria_MN194"                 
## [158] "Junodia_amoena_MN286"                
## [159] "Kalotermes_flavicollis_IS027"        
## [160] "Kongobatha_diademata_MN306"          
## [161] "Leptocola_stanleyana_MN284"          
## [162] "Leptomantella_albella_MN095"         
## [163] "Leptomantella_albella_MN319"         
## [164] "Leptomantella_sp._MN108"             
## [165] "Leptomantella_sp._MN198"             
## [166] "Leptomantella_sp._MN322"             
## [167] "Ligaria_brevicollis_ignota_MN138"    
## [168] "Ligaria_brevicollis_ignota_MN211"    
## [169] "Ligaria_brevicollis_ignota_MN303"    
## [170] "Ligariella_gracilis_MN041"           
## [171] "Ligariella_trigonalis_MN260"         
## [172] "Ligentella_beieri_MN299"             
## [173] "Ligentella_zairensis_MN213"          
## [174] "Litaneutria_minor_MN045"             
## [175] "Liturgusa_maya_MN145"                
## [176] "Liturgusa_sp._MN116"                 
## [177] "Macromantis_nicaraguae_MN144"        
## [178] "Macromusonia_conspersa_MN126"        
## [179] "Macrotermes_subhyalinus_IS095"       
## [180] "Mantis_religiosa_MN001"              
## [181] "Mantis_religiosa_MN247"              
## [182] "Mantoida_schraderi_MN009"            
## [183] "Mantoida_sp._MN109"                  
## [184] "Mantoida_sp._MN110"                  
## [185] "Mantoida_sp._MN179"                  
## [186] "Mantoida_sp._MN180"                  
## [187] "Mastotermes_darwiniensis_IS034"      
## [188] "Metallyticus_fallax_MN157A"          
## [189] "Metallyticus_fallax_MN157B"          
## [190] "Metallyticus_splendidus_MN156A"      
## [191] "Metallyticus_splendidus_MN156B"      
## [192] "Metilia_boliviana_MN311"             
## [193] "Metilia_brunnerii_MN146"             
## [194] "Microphotina_vitripennis_MN271"      
## [195] "Miomantis_aurea_MN228"               
## [196] "Miomantis_paykullii_MN245"           
## [197] "Miomantis_sp._MN181"                 
## [198] "Miomantis_sp._MN191"                 
## [199] "Miomantis_sp._MN196"                 
## [200] "Miromantis_mirandula_MN323"          
## [201] "Miromantis_mirandula_MN338"          
## [202] "Musoniella_sp._MN122"                
## [203] "Musoniella_sp._MN137"                
## [204] "Myrcinus_tuberosus_MN257"            
## [205] "Namamantis_nigropunctata_MN302"      
## [206] "Neomantis_hyalina_MN052"             
## [207] "Nilomantis_edmundsi_MN244"           
## [208] "Nothogalepsus_planivertex_MN186"     
## [209] "Nothogalepsus_sp._MN174"             
## [210] "Oligonicella_punctulata_MN049"       
## [211] "Oligonicella_scudderi_MN057"         
## [212] "Omomantis_zebrata_MN276"             
## [213] "Oromantis_sp._MN152"                 
## [214] "Orthodera_novaezealandiae_MN007"     
## [215] "Orthodera_sp._MN033"                 
## [216] "Orthoderella_ornata_MN060"           
## [217] "Orthoderella_ornata_MN129"           
## [218] "Otomantis_rendalli_MN290"            
## [219] "Otomantis_scutigera_MN155"           
## [220] "Otomantis_sp._MN161"                 
## [221] "Oxyophthalma_engaea_MN200"           
## [222] "Oxyothespis_sp._MN283"               
## [223] "Oxypiloidea_subcornuta_MN281"        
## [224] "Oxypiloidea_tridens_MN289"           
## [225] "Oxypilus_hamatus_MN246"              
## [226] "Oxypilus_masutus_MN205"              
## [227] "Oxypilus_nigericus_MN287"            
## [228] "Oxypilus_sp._MN154"                  
## [229] "Oxypilus_transvalensis_MN172"        
## [230] "Panurgica_compressicollis_MN232"     
## [231] "Panurgica_fratercula_MN227"          
## [232] "Paragalepsus_toganus_MN243"          
## [233] "Paraoxypilus_tasmaniensis_MN022"     
## [234] "Paraoxypilus_verreauxii_MN008"       
## [235] "Parasphendale_sp._MN195"             
## [236] "Parastagmatoptera_sp._MN028"         
## [237] "Parastagmatoptera_sp._MN125"         
## [238] "Parastagmatoptera_sp._MN151"         
## [239] "Parathespis_humbertiana_MN263"       
## [240] "Photina_sp._MN295"                   
## [241] "Phyllothelys_decipiens_MN101"        
## [242] "Phyllothelys_decipiens_MN329"        
## [243] "Phyllothelys_westwoodi_MN076"        
## [244] "Phyllovates_cingulata_MN214"         
## [245] "Plistospilota_guineensis_MN236"      
## [246] "Polyspilota_aeruginosa_MN167"        
## [247] "Polyspilota_aeruginosa_MN248"        
## [248] "Popa_spurca_MN133"                   
## [249] "Popa_undata_MN164"                   
## [250] "Prohierodula_ornatipennis_MN249"     
## [251] "Pseudocreobotra_occellata_MN231"     
## [252] "Pseudoharpax_uganda_MN170"           
## [253] "Pseudomantis_albofimbriata_MN314"    
## [254] "Pseudomiopteryx_guyanensis_MN114"    
## [255] "Pseudothespis_meghalayensis_MN206"   
## [256] "Pseudovates_denticulata_MN312"       
## [257] "Psychomantis_borneensis_MN320"       
## [258] "Pyrgomantis_jonesi_MN224"            
## [259] "Pyrgomantis_nasuta_MN184"            
## [260] "Raptrix_fusca_MN113"                 
## [261] "Raptrix_persiara_MN203"              
## [262] "Raptrix_perspicua_MN177"             
## [263] "Reticulitermes_santonensis_IS054"    
## [264] "Rhomantis_moultoni_MN106"            
## [265] "Rhombodera_basalis_MN344"            
## [266] "Rogermantis_royi_MN301"              
## [267] "Sceptuchus_simplex_MN104"            
## [268] "Schizocephala_bicornis_MN065"        
## [269] "Sibylla_dives_MN285"                 
## [270] "Sibylla_operosa_MN226"               
## [271] "Sibylla_pretiosa_MN135"              
## [272] "Sibylla_pretiosa_MN173"              
## [273] "Sphodromantis_lineola_MN015"         
## [274] "Sphodromantis_viridis_MN013"         
## [275] "Sphodropoda_moesta_MN275"            
## [276] "Stagmatoptera_sp._MN029"             
## [277] "Stagmatoptera_sp._MN117"             
## [278] "Stagmomantis_carolina_MN023"         
## [279] "Stagmomantis_sp._MN027"              
## [280] "Stagmomantis_sp._MN149"              
## [281] "Stagmomantis_sp._MN150"              
## [282] "Stagmomantis_sp._MN278"              
## [283] "Stagmomantis_sp._MN279"              
## [284] "Stagmomantis_vicina_MN024"           
## [285] "Statilia_apicalis_MN048"             
## [286] "Statilia_apicalis_MN050"             
## [287] "Statilia_maculata_MN062"             
## [288] "Statilia_maculata_MN336"             
## [289] "Statilia_maculata_MN346"             
## [290] "Statilia_nemoralis_MN078"            
## [291] "Statilia_sp._MN255"                  
## [292] "Stenomantis_novaeguineae_MN030"      
## [293] "Stenomantis_novaeguineae_MN037"      
## [294] "Stenomantis_novaeguineae_MN038"      
## [295] "Stenomantis_novaeguineae_MN039"      
## [296] "Stenopyga_ziela_MN235"               
## [297] "Stenotoxodera_porioni_MN090"         
## [298] "Supella_longipalpa_BL138"            
## [299] "Tamolanica_tamolana_MN020"           
## [300] "Tarachina_occidentalis_MN237"        
## [301] "Tarachina_sp._MN140"                 
## [302] "Tarachodes_afzelii_MN158"            
## [303] "Tarachodes_afzelii_MN238"            
## [304] "Tarachodes_dissimulator_MN233"       
## [305] "Tarachodes_sp._MN134"                
## [306] "Tarachodula_pantherina_MN309"        
## [307] "Tarachomantis_caldwellii_MN310"      
## [308] "Taumantis_ehrmannii_MN163"           
## [309] "Tenodera_costalis_MN025"             
## [310] "Termes_hispaniolae_IS191"            
## [311] "Theopompella_chopardi_MN230"         
## [312] "Theopropus_elegans_MN166"            
## [313] "Thesprotia_graminis_MN058"           
## [314] "Thesprotia_macilenta_MN313"          
## [315] "Thesprotia_sp._MN121"                
## [316] "Thesprotiella_peruana_MN269"         
## [317] "Thesprotiella_sp._MN252"             
## [318] "Thrinaconyx_fumosa_MN046"            
## [319] "Thrinaconyx_fumosa_MN086"            
## [320] "Thrinaconyx_kirschianus_MN216"       
## [321] "Toxoderopsis_taurus_MN068"           
## [322] "Tropidomantis_tenera_MN096"          
## [323] "Tropidomantis_tenera_MN107"          
## [324] "Tropidomantis_tenera_MN212"          
## [325] "Tropidomantis_tenera_MN341"          
## [326] "Tylomantis_sp._MN047"                
## [327] "Vates_pectinacornis_MN014"           
## [328] "Vates_sp._MN118"                     
## [329] "Vates_sp._MN119"                     
## [330] "Vates_sp._MN128"                     
## [331] "Xanthomantis_mantispoides_MN099"     
## [332] "Xanthomantis_mantispoides_MN335"     
## [333] "Xystropeltis_sp._MN268"              
## [334] "Yersinia_mexicana_MN273"             
## 
## $data_not_tree
## character(0)
```

```
#remove tips from tree with no data
tree.1<-drop.tip(tree, foo$tree_not_data)
plot(tree.1)
```

```
overlap <- name.check(tree.1, data=data.1)
overlap
```

```
## [1] "OK"
```

```
is_tip <- tree.1$edge[,2] <= length(tree.1$tip.label)
ordered_tips <- tree.1$edge[is_tip, 2]
tree.1$tip.label[ordered_tips]
```

```
##  [1] "Phyllocrania_paradoxa_MN011"     "Hymenopus_coronatus_MN010"      
##  [3] "Theopropus_elegans_MN094"        "Pseudocreobotra_occellata_MN017"
##  [5] "Euchomenella_sp._MN091"          "Deroplatys_truncata_MN349"      
##  [7] "Tenodera_aridifolia_MN002"       "Stagmomantis_limbata_MN031"     
##  [9] "Oxyopsis_sp._MN294"              "Phyllovates_chlorophaea_MN026"
```

```
data.1<-data.1[tree.1$tip.label,]
attach(data.1)
name.check(tree.1, data.1)
```

```
## [1] "OK"
```

```
inv.phylo<-inverseA(tree.1,nodes="TIPS",scale=FALSE)
prior2<-list(G=list(G1=list(V=1,nu=0.02),G2=list(V=1,nu=0.02)),
   R=list(V=1,nu=0.02))

  
#morphology data
morpho_df <- data.frame(max.data$Body_length, max.data$Tibia_length, max.data$Femur_Length, max.data$Coxa_length)

#total data frame for final PCA includes 16 variables in this study
total_df <- data.frame(max.data$Coxa_LV, max.data$Femur_LV, max.data$Coxa_AV, max.data $Femur_AV, max.data$abs_tibia_AV, max.data$abs_tibia_LV, max.data$Coxa.start, max.data$Approach.Time, max.data$PP_angle_st, max.data$PP_dist_cm_st, max.data$BD_veloc_cm.s, max.data$BD_dist_cm, max.data$TTPC.Sweep, max.data$CF_lateral_disp, max.data$FT_lateral_disp, max.data$tib_lateral_disp)
```

# Analyses

## Principal Component Analysis on morphology based on 3 maximum attempts

```
#morpho
pca_morpho <- princomp(morpho_df, cor = TRUE)
max.data$mPC1<-pca_morpho$scores[,1]  
max.data$mPC2<-pca_morpho$scores[,2]
summary(pca_morpho)
```

```
## Importance of components:
##                           Comp.1    Comp.2     Comp.3     Comp.4
## Standard deviation     1.6493495 1.0716655 0.27952382 0.23031665
## Proportion of Variance 0.6800884 0.2871167 0.01953339 0.01326144
## Cumulative Proportion  0.6800884 0.9672052 0.98673856 1.00000000
```

## PCA on 16 kinematic variable based on 3 maximum attempts

```
pca_total <- princomp(total_df, cor = TRUE)
sum.pca<-summary(pca_total)

#loadings for each PC axis
#pca_total$loadings
kine_pca<-pca_total$loadings
#unblock following to lines to save PCA loadings and summary
#write.csv(kine_pca,"kine_pca.csv")
#write.csv(sum.pca,"sum_pca.csv")

bstick_total <- screeplot(x = pca_total, bstick = TRUE, type = c("barplot", "lines"),npcs = min(10, length(pca_total$sdev)), ptype = "o", bst.col = "red", bst.lty = "solid", xlab = "Component", ylab = "Inertia", main = deparse(substitute(pca_total)))
```

```
bstick_total
```

```
## $x
##  [1]  0.7  1.9  3.1  4.3  5.5  6.7  7.9  9.1 10.3 11.5
## 
## $y
##  [1] 4.1456349 2.5892434 2.4067130 1.5233005 1.2457284 0.9876355 0.7624338
##  [8] 0.5364830 0.4021174 0.3621050
## 
## $xlab
## NULL
## 
## $ylab
## NULL
```

```
max.data$total_PC1<-pca_total$scores[,1]
max.data$total_PC2<-pca_total$scores[,2]
max.data$total_PC3<-pca_total$scores[,3]
```

### PCA summary statistics and loadings for 16 kinematic traits

```
#summary statistics to get proportional variances
sum.pca
```

```
## Importance of components:
##                           Comp.1    Comp.2    Comp.3     Comp.4     Comp.5
## Standard deviation     2.0360832 1.6091126 1.5513584 1.23422059 1.11612203
## Proportion of Variance 0.2591022 0.1618277 0.1504196 0.09520628 0.07785802
## Cumulative Proportion  0.2591022 0.4209299 0.5713495 0.66655573 0.74441376
##                            Comp.6     Comp.7     Comp.8     Comp.9    Comp.10
## Standard deviation     0.99379851 0.87317456 0.73245001 0.63412728 0.60175158
## Proportion of Variance 0.06172722 0.04765211 0.03353019 0.02513234 0.02263156
## Cumulative Proportion  0.80614097 0.85379309 0.88732328 0.91245561 0.93508717
##                           Comp.11    Comp.12    Comp.13     Comp.14     Comp.15
## Standard deviation     0.52422907 0.47929218 0.43445854 0.381677384 0.327798609
## Proportion of Variance 0.01717601 0.01435756 0.01179714 0.009104852 0.006715746
## Cumulative Proportion  0.95226318 0.96662074 0.97841788 0.987522735 0.994238480
##                           Comp.16
## Standard deviation     0.30361870
## Proportion of Variance 0.00576152
## Cumulative Proportion  1.00000000
```

```
#loadings for each PC axis
pca_total$loadings
```

```
## 
## Loadings:
##                           Comp.1 Comp.2 Comp.3 Comp.4 Comp.5 Comp.6 Comp.7
## max.data.Coxa_LV                  0.402  0.296  0.180                0.298
## max.data.Femur_LV         -0.314  0.215  0.115  0.170 -0.368 -0.310       
## max.data.Coxa_AV          -0.196  0.337                0.502  0.310       
## max.data.Femur_AV         -0.368  0.248         0.267 -0.156        -0.109
## max.data.abs_tibia_AV     -0.230  0.259 -0.188 -0.373                0.453
## max.data.abs_tibia_LV     -0.262  0.238 -0.262 -0.249 -0.188         0.364
## max.data.Coxa.start       -0.179        -0.397 -0.112 -0.362  0.130 -0.486
## max.data.Approach.Time     0.144 -0.130  0.129  0.374 -0.482  0.285  0.418
## max.data.PP_angle_st       0.155        -0.293 -0.120 -0.159  0.726       
## max.data.PP_dist_cm_st     0.182  0.282  0.280 -0.306 -0.355 -0.142 -0.193
## max.data.BD_veloc_cm.s            0.458  0.185  0.205         0.139 -0.178
## max.data.BD_dist_cm        0.342  0.296  0.138 -0.218               -0.214
## max.data.TTPC.Sweep        0.347               -0.385                0.150
## max.data.CF_lateral_disp   0.307        -0.281  0.330        -0.164       
## max.data.FT_lateral_disp   0.299  0.190 -0.362  0.210        -0.219       
## max.data.tib_lateral_disp  0.246  0.222 -0.425  0.102  0.113 -0.210       
##                           Comp.8 Comp.9 Comp.10 Comp.11 Comp.12 Comp.13 Comp.14
## max.data.Coxa_LV           0.447         0.449           0.270   0.283   0.157 
## max.data.Femur_LV          0.329        -0.243  -0.154  -0.170           0.100 
## max.data.Coxa_AV                        -0.352   0.191   0.350  -0.371  -0.111 
## max.data.Femur_AV                 0.117 -0.373  -0.221           0.126   0.180 
## max.data.abs_tibia_AV     -0.274 -0.321 -0.159   0.101           0.497  -0.106 
## max.data.abs_tibia_LV             0.387  0.295   0.113          -0.540   0.104 
## max.data.Coxa.start                      0.210           0.541   0.188         
## max.data.Approach.Time    -0.302 -0.278 -0.138           0.287  -0.206         
## max.data.PP_angle_st       0.355                        -0.401                 
## max.data.PP_dist_cm_st     0.182 -0.292          0.266          -0.208  -0.438 
## max.data.BD_veloc_cm.s    -0.519  0.249  0.196  -0.191  -0.218   0.101  -0.361 
## max.data.BD_dist_cm       -0.250                 0.126                   0.742 
## max.data.TTPC.Sweep               0.458 -0.390  -0.388   0.378                 
## max.data.CF_lateral_disp          0.389 -0.243   0.641           0.214         
## max.data.FT_lateral_disp         -0.130  0.156  -0.369   0.134                 
## max.data.tib_lateral_disp        -0.342 -0.122  -0.163          -0.175         
##                           Comp.15 Comp.16
## max.data.Coxa_LV           0.160   0.101 
## max.data.Femur_LV         -0.580   0.120 
## max.data.Coxa_AV          -0.202         
## max.data.Femur_AV          0.619  -0.228 
## max.data.abs_tibia_AV     -0.109         
## max.data.abs_tibia_LV                    
## max.data.Coxa.start                0.138 
## max.data.Approach.Time                   
## max.data.PP_angle_st      -0.103         
## max.data.PP_dist_cm_st     0.257  -0.170 
## max.data.BD_veloc_cm.s    -0.143   0.197 
## max.data.BD_dist_cm       -0.119  -0.105 
## max.data.TTPC.Sweep                0.119 
## max.data.CF_lateral_disp                 
## max.data.FT_lateral_disp  -0.158  -0.639 
## max.data.tib_lateral_disp  0.227   0.622 
## 
##                Comp.1 Comp.2 Comp.3 Comp.4 Comp.5 Comp.6 Comp.7 Comp.8 Comp.9
## SS loadings     1.000  1.000  1.000  1.000  1.000  1.000  1.000  1.000  1.000
## Proportion Var  0.063  0.062  0.062  0.062  0.062  0.063  0.062  0.062  0.062
## Cumulative Var  0.063  0.125  0.187  0.250  0.312  0.375  0.437  0.500  0.562
##                Comp.10 Comp.11 Comp.12 Comp.13 Comp.14 Comp.15 Comp.16
## SS loadings      1.000   1.000   1.000   1.000   1.000   1.000   1.000
## Proportion Var   0.063   0.062   0.062   0.062   0.062   0.062   0.063
## Cumulative Var   0.625   0.687   0.750   0.812   0.875   0.937   1.000
```

## PGLMM effects camouflage on morphology

```
#aggregate by individual
data.2<-aggregate(max.data[,16:100], by=list(max.data$Species,max.data$Phylo,max.data$Mantis,max.data$Ecomorph),
FUN=mean, na.rm=TRUE)   
colnames(data.2)[1] ="species"
colnames(data.2)[2] ="phylo"
colnames(data.2)[3] ="Mantis"
colnames(data.2)[4] ="Ecomorph"
   
data.2$spec_mean_mPC1<-sapply(split(data.2$mPC1,data.2$phylo),mean)[data.2$phylo]
data.2$within_spec_mPC1<-data.2$mPC1-data.2$spec_mean_mPC1       

data.2$spec_mean_mPC2<-sapply(split(data.2$mPC2,data.2$phylo),mean)[data.2$phylo]
data.2$within_spec_mPC2<-data.2$mPC2-data.2$spec_mean_mPC2
```

### Morpho PC1 reults

```
#morpho PC1   
model_mPC1<-MCMCglmm(mPC1~Ecomorph,
    random=~phylo+species,family="gaussian",
    ginverse=list(phylo=inv.phylo$Ainv),prior=prior2,data=data.2,
    nitt=1000000,burnin=1000,thin=500,scale=TRUE,verbose=FALSE)
  summary(model_mPC1)
```

```
## 
##  Iterations = 1001:999501
##  Thinning interval  = 500
##  Sample size  = 1998 
## 
##  DIC: 55.31242 
## 
##  G-structure:  ~phylo
## 
##       post.mean l-95% CI u-95% CI eff.samp
## phylo     19.81 0.002762    66.76     1675
## 
##                ~species
## 
##         post.mean l-95% CI u-95% CI eff.samp
## species     1.258 0.002771     4.56     1998
## 
##  R-structure:  ~units
## 
##       post.mean l-95% CI u-95% CI eff.samp
## units    0.1501  0.08884   0.2167     2247
## 
##  Location effects: mPC1 ~ Ecomorph 
## 
##                    post.mean l-95% CI u-95% CI eff.samp pMCMC
## (Intercept)           -0.269   -2.400    2.134     1998 0.776
## EcomorphFlower        -1.272   -4.400    1.808     1998 0.387
## EcomorphGeneralist     1.442   -1.763    4.439     1661 0.297
## EcomorphStick          1.943   -1.508    5.082     1662 0.193
```

```
m<-emmeans(model_mPC1,specs=~Ecomorph,data=data.2)
summary(m,freq=TRUE)
```

```
##  Ecomorph   emmean   SE  df asymp.LCL asymp.UCL
##  Dead Leaf  -0.269 1.14 Inf    -2.510     1.972
##  Flower     -1.541 1.12 Inf    -3.730     0.648
##  Generalist  1.173 1.11 Inf    -1.001     3.347
##  Stick       1.674 1.16 Inf    -0.605     3.952
## 
## Confidence level used: 0.95
```

```
summary(pairs(m),freq=TRUE)
```

```
##  contrast               estimate   SE  df z.ratio p.value
##  Dead Leaf - Flower        1.272 1.58 Inf   0.804  0.8528
##  Dead Leaf - Generalist   -1.442 1.57 Inf  -0.918  0.7951
##  Dead Leaf - Stick        -1.943 1.61 Inf  -1.208  0.6214
##  Flower - Generalist      -2.714 1.54 Inf  -1.761  0.2922
##  Flower - Stick           -3.215 1.61 Inf  -2.002  0.1870
##  Generalist - Stick       -0.501 1.41 Inf  -0.354  0.9848
## 
## P value adjustment: tukey method for comparing a family of 4 estimates
```

```
#no differences
#lambda Morpho PC 1
lambda <- model_mPC1$VCV[,'phylo']/
 (model_mPC1$VCV[,'phylo']+model_mPC1$VCV[,'species']+
 model_mPC1$VCV[,'units'])
#lambda Morpho PC 1
mean(lambda)
```

```
## [1] 0.6889533
```

### Morpho PC 2 results

```
#Morpho PC2
model_mPC2<-MCMCglmm(mPC2~Ecomorph,
    random=~phylo+species,family="gaussian",
    ginverse=list(phylo=inv.phylo$Ainv),prior=prior2,data=data.2,
    nitt=1000000,burnin=1000,thin=500,scale=TRUE,verbose=FALSE)
  summary(model_mPC2)
```

```
## 
##  Iterations = 1001:999501
##  Thinning interval  = 500
##  Sample size  = 1998 
## 
##  DIC: 77.86885 
## 
##  G-structure:  ~phylo
## 
##       post.mean l-95% CI u-95% CI eff.samp
## phylo      5.88  0.00237    27.29     1994
## 
##                ~species
## 
##         post.mean l-95% CI u-95% CI eff.samp
## species    0.7817 0.003364    2.178     1680
## 
##  R-structure:  ~units
## 
##       post.mean l-95% CI u-95% CI eff.samp
## units     0.238   0.1389     0.35     1998
## 
##  Location effects: mPC2 ~ Ecomorph 
## 
##                    post.mean l-95% CI u-95% CI eff.samp pMCMC
## (Intercept)           0.2609  -1.3501   1.7549     1998 0.713
## EcomorphFlower       -0.7878  -3.0187   1.2679     1998 0.385
## EcomorphGeneralist   -0.7214  -2.8692   1.1842     1998 0.411
## EcomorphStick         0.8649  -1.2833   3.1606     1998 0.374
```

```
m<-emmeans(model_mPC2,specs=~Ecomorph,data=data.2)
summary(m,freq=TRUE)
```

```
##  Ecomorph   emmean    SE  df asymp.LCL asymp.UCL
##  Dead Leaf   0.261 0.808 Inf    -1.323     1.844
##  Flower     -0.527 0.698 Inf    -1.895     0.841
##  Generalist -0.461 0.702 Inf    -1.836     0.915
##  Stick       1.126 0.807 Inf    -0.457     2.708
## 
## Confidence level used: 0.95
```

```
summary(pairs(m),freq=TRUE)
```

```
##  contrast               estimate    SE  df z.ratio p.value
##  Dead Leaf - Flower       0.7878 1.060 Inf   0.743  0.8795
##  Dead Leaf - Generalist   0.7214 1.044 Inf   0.691  0.9006
##  Dead Leaf - Stick       -0.8649 1.133 Inf  -0.763  0.8709
##  Flower - Generalist     -0.0664 0.967 Inf  -0.069  0.9999
##  Flower - Stick          -1.6527 1.055 Inf  -1.567  0.3975
##  Generalist - Stick      -1.5863 0.984 Inf  -1.612  0.3720
## 
## P value adjustment: tukey method for comparing a family of 4 estimates
```

```
#no differences
lambda <- model_mPC2$VCV[,'phylo']/
 (model_mPC2$VCV[,'phylo']+model_mPC2$VCV[,'species']+
 model_mPC2$VCV[,'units'])
#lambda Morpho PC 2
mean(lambda)
```

```
## [1] 0.4906104
```

## PGLMM effects camouflage and morphology PC 1-2 on Kinematic PC 1-3

### Kinematic PC 1

```
model_kPC1<-MCMCglmm(total_PC1~Ecomorph+spec_mean_mPC1+within_spec_mPC1+spec_mean_mPC2+within_spec_mPC2,
    random=~phylo+species,family="gaussian",
    ginverse=list(phylo=inv.phylo$Ainv),prior=prior2,data=data.2,
    nitt=1000000,burnin=1000,thin=500,scale=TRUE,verbose=FALSE)
  summary(model_kPC1)
```

```
## 
##  Iterations = 1001:999501
##  Thinning interval  = 500
##  Sample size  = 1998 
## 
##  DIC: 135.7718 
## 
##  G-structure:  ~phylo
## 
##       post.mean l-95% CI u-95% CI eff.samp
## phylo     24.06 0.002291    96.22     1761
## 
##                ~species
## 
##         post.mean l-95% CI u-95% CI eff.samp
## species     2.147 0.002675    7.288     1998
## 
##  R-structure:  ~units
## 
##       post.mean l-95% CI u-95% CI eff.samp
## units    0.7434   0.4502    1.114     1998
## 
##  Location effects: total_PC1 ~ Ecomorph + spec_mean_mPC1 + within_spec_mPC1 + spec_mean_mPC2 + within_spec_mPC2 
## 
##                    post.mean l-95% CI u-95% CI eff.samp  pMCMC  
## (Intercept)         -0.93671 -3.95001  1.71855     1998 0.3994  
## EcomorphFlower      -0.35829 -5.07319  4.23652     1727 0.8338  
## EcomorphGeneralist   2.54833 -2.09469  6.22244     1998 0.1582  
## EcomorphStick        1.55018 -4.11583  7.03991     1998 0.5055  
## spec_mean_mPC1       0.07549 -1.57124  1.43474     1998 0.9099  
## within_spec_mPC1     1.06792 -0.03293  2.24956     1878 0.0621 .
## spec_mean_mPC2       0.07397 -1.87380  2.10127     1998 0.9520  
## within_spec_mPC2     0.63147 -0.23900  1.59703     1998 0.1622  
## ---
## Signif. codes:  0 '***' 0.001 '**' 0.01 '*' 0.05 '.' 0.1 ' ' 1
```

```
lambda <- model_kPC1$VCV[,'phylo']/
 (model_kPC1$VCV[,'phylo']+model_kPC1$VCV[,'species']+
 model_kPC1$VCV[,'units'])
#lambda full model
mean(lambda)
```

```
## [1] 0.5098485
```

```
model_kPC1.1<-MCMCglmm(mPC1~Ecomorph,
    random=~phylo+species,family="gaussian",
    ginverse=list(phylo=inv.phylo$Ainv),prior=prior2,data=data.2,
    nitt=1000000,burnin=1000,thin=500, scale=TRUE,verbose=FALSE)
  summary(model_kPC1.1)
```

```
## 
##  Iterations = 1001:999501
##  Thinning interval  = 500
##  Sample size  = 1998 
## 
##  DIC: 55.28175 
## 
##  G-structure:  ~phylo
## 
##       post.mean l-95% CI u-95% CI eff.samp
## phylo     20.64 0.002295    69.49     2171
## 
##                ~species
## 
##         post.mean l-95% CI u-95% CI eff.samp
## species     1.261 0.001859    4.324     1998
## 
##  R-structure:  ~units
## 
##       post.mean l-95% CI u-95% CI eff.samp
## units    0.1503  0.08945   0.2181     1998
## 
##  Location effects: mPC1 ~ Ecomorph 
## 
##                    post.mean l-95% CI u-95% CI eff.samp pMCMC
## (Intercept)          -0.3129  -2.8769   1.8170     1998 0.753
## EcomorphFlower       -1.2636  -4.5297   1.8389     2171 0.378
## EcomorphGeneralist    1.5038  -1.6185   4.8010     1998 0.295
## EcomorphStick         1.9895  -1.1394   5.3929     1998 0.184
```

```
m<-emmeans(model_kPC1.1,specs=~Ecomorph,data=data.2)
summary(m,freq=TRUE)
```

```
##  Ecomorph   emmean   SE  df asymp.LCL asymp.UCL
##  Dead Leaf  -0.313 1.20 Inf    -2.667     2.042
##  Flower     -1.576 1.15 Inf    -3.821     0.668
##  Generalist  1.191 1.10 Inf    -0.971     3.353
##  Stick       1.677 1.17 Inf    -0.620     3.974
## 
## Confidence level used: 0.95
```

```
summary(pairs(m),freq=TRUE)
```

```
##  contrast               estimate   SE  df z.ratio p.value
##  Dead Leaf - Flower        1.264 1.61 Inf   0.783  0.8621
##  Dead Leaf - Generalist   -1.504 1.65 Inf  -0.911  0.7991
##  Dead Leaf - Stick        -1.990 1.63 Inf  -1.219  0.6148
##  Flower - Generalist      -2.767 1.57 Inf  -1.761  0.2924
##  Flower - Stick           -3.253 1.65 Inf  -1.976  0.1970
##  Generalist - Stick       -0.486 1.42 Inf  -0.342  0.9863
## 
## P value adjustment: tukey method for comparing a family of 4 estimates
```

```
 lambda <- model_kPC1.1$VCV[,'phylo']/
 (model_kPC1.1$VCV[,'phylo']+model_kPC1.1$VCV[,'species']+
 model_kPC1.1$VCV[,'units'])
#lambda camouflage only
 mean(lambda)
```

```
## [1] 0.7006017
```

#### AICc comparison for K PC1 models

```
AICc(model_kPC1,model_kPC1.1)
```

```
##              df      AICc
## model_kPC1   11 152.46713
## model_kPC1.1  7  61.38435
```

### Kinematic PC 2

```
model_kPC2<-MCMCglmm(total_PC2~Ecomorph+spec_mean_mPC1+within_spec_mPC1+spec_mean_mPC2+within_spec_mPC2,
    random=~phylo+species,family="gaussian",
    ginverse=list(phylo=inv.phylo$Ainv),prior=prior2,data=data.2,
    nitt=1000000,burnin=1000,thin=500,scale=TRUE,verbose=FALSE)
  summary(model_kPC2)
```

```
## 
##  Iterations = 1001:999501
##  Thinning interval  = 500
##  Sample size  = 1998 
## 
##  DIC: 141.1088 
## 
##  G-structure:  ~phylo
## 
##       post.mean l-95% CI u-95% CI eff.samp
## phylo     3.384 0.002003    14.52     1998
## 
##                ~species
## 
##         post.mean l-95% CI u-95% CI eff.samp
## species    0.3641  0.00177    1.264     1998
## 
##  R-structure:  ~units
## 
##       post.mean l-95% CI u-95% CI eff.samp
## units    0.8566    0.511    1.264     2208
## 
##  Location effects: total_PC2 ~ Ecomorph + spec_mean_mPC1 + within_spec_mPC1 + spec_mean_mPC2 + within_spec_mPC2 
## 
##                    post.mean l-95% CI u-95% CI eff.samp  pMCMC  
## (Intercept)         -1.25003 -2.49857 -0.10929     2255 0.0440 *
## EcomorphFlower       2.05776 -0.29303  4.08253     1871 0.0581 .
## EcomorphGeneralist   0.97887 -0.91884  2.81436     1998 0.2032  
## EcomorphStick        1.54629 -0.78935  4.29791     2133 0.1742  
## spec_mean_mPC1       0.12224 -0.57797  0.71792     2153 0.6266  
## within_spec_mPC1     1.19055 -0.02261  2.39651     2134 0.0591 .
## spec_mean_mPC2       0.26594 -0.59959  1.33772     2164 0.5015  
## within_spec_mPC2     0.31822 -0.68451  1.24854     1998 0.5045  
## ---
## Signif. codes:  0 '***' 0.001 '**' 0.01 '*' 0.05 '.' 0.1 ' ' 1
```

```
m<-emmeans(model_kPC2,specs=~Ecomorph,data=data.2)#working, but can't get p-value
summary(m,freq=TRUE)
```

```
##  Ecomorph   emmean    SE  df asymp.LCL asymp.UCL
##  Dead Leaf  -1.256 0.636 Inf     -2.50   -0.0106
##  Flower      0.801 0.934 Inf     -1.03    2.6310
##  Generalist -0.278 0.614 Inf     -1.48    0.9255
##  Stick       0.290 1.166 Inf     -2.00    2.5752
## 
## Confidence level used: 0.95
```

```
summary(pairs(m),freq=TRUE)
```

```
##  contrast               estimate    SE  df z.ratio p.value
##  Dead Leaf - Flower       -2.058 1.052 Inf  -1.955  0.2052
##  Dead Leaf - Generalist   -0.979 0.904 Inf  -1.083  0.6999
##  Dead Leaf - Stick        -1.546 1.347 Inf  -1.148  0.6598
##  Flower - Generalist       1.079 1.204 Inf   0.896  0.8068
##  Flower - Stick            0.511 1.905 Inf   0.268  0.9932
##  Generalist - Stick       -0.567 1.180 Inf  -0.481  0.9634
## 
## P value adjustment: tukey method for comparing a family of 4 estimates
```

```
lambda <- model_kPC2$VCV[,'phylo']/
 (model_kPC2$VCV[,'phylo']+model_kPC2$VCV[,'species']+
 model_kPC2$VCV[,'units'])
#lambda full model
mean(lambda)
```

```
## [1] 0.4002669
```

```
#use this model better fit based on AICc
  model_kPC2.1<-MCMCglmm(total_PC2~Ecomorph,
    random=~phylo+species,family="gaussian",
    ginverse=list(phylo=inv.phylo$Ainv),prior=prior2,data=data.2,
    nitt=1000000,burnin=1000,thin=500,scale=TRUE,verbose=FALSE)
  summary(model_kPC2.1)
```

```
## 
##  Iterations = 1001:999501
##  Thinning interval  = 500
##  Sample size  = 1998 
## 
##  DIC: 141.4683 
## 
##  G-structure:  ~phylo
## 
##       post.mean l-95% CI u-95% CI eff.samp
## phylo     1.819 0.001919    7.909     1998
## 
##                ~species
## 
##         post.mean l-95% CI u-95% CI eff.samp
## species    0.1617 0.002499   0.6193     1998
## 
##  R-structure:  ~units
## 
##       post.mean l-95% CI u-95% CI eff.samp
## units    0.9296    0.559    1.361     1998
## 
##  Location effects: total_PC2 ~ Ecomorph 
## 
##                    post.mean l-95% CI u-95% CI eff.samp pMCMC  
## (Intercept)          -1.2300  -2.1104  -0.1974     1878 0.019 *
## EcomorphFlower        1.7012   0.4407   3.0240     1964 0.021 *
## EcomorphGeneralist    0.9425  -0.4088   2.1225     1998 0.127  
## EcomorphStick         2.0473   0.6908   3.3636     1998 0.010 *
## ---
## Signif. codes:  0 '***' 0.001 '**' 0.01 '*' 0.05 '.' 0.1 ' ' 1
```

```
#significant if just ecomorph
m<-emmeans(model_kPC2.1,specs=~Ecomorph,data=data.2)#working, but can't get p-value
summary(m,freq=TRUE)
```

```
##  Ecomorph   emmean    SE  df asymp.LCL asymp.UCL
##  Dead Leaf  -1.230 0.494 Inf    -2.198    -0.262
##  Flower      0.471 0.455 Inf    -0.420     1.362
##  Generalist -0.288 0.418 Inf    -1.106     0.531
##  Stick       0.817 0.482 Inf    -0.127     1.762
## 
## Confidence level used: 0.95
```

```
summary(pairs(m),freq=TRUE)
```

```
##  contrast               estimate    SE  df z.ratio p.value
##  Dead Leaf - Flower       -1.701 0.661 Inf  -2.573  0.0495
##  Dead Leaf - Generalist   -0.942 0.637 Inf  -1.480  0.4497
##  Dead Leaf - Stick        -2.047 0.677 Inf  -3.026  0.0132
##  Flower - Generalist       0.759 0.623 Inf   1.218  0.6151
##  Flower - Stick           -0.346 0.649 Inf  -0.534  0.9509
##  Generalist - Stick       -1.105 0.611 Inf  -1.807  0.2700
## 
## P value adjustment: tukey method for comparing a family of 4 estimates
```

```
lambda <- model_kPC2.1$VCV[,'phylo']/
 (model_kPC2.1$VCV[,'phylo']+model_kPC2.1$VCV[,'species']+
 model_kPC2.1$VCV[,'units'])
#lambbda camouflage only
mean(lambda)
```

```
## [1] 0.331149
```

#### AICc comparison for K PC2 models

```
AICc(model_kPC2,model_kPC2.1)
```

```
##              df     AICc
## model_kPC2   11 159.0100
## model_kPC2.1  7 150.7321
```

### Kinematic PC 3

```
model_kPC3<-MCMCglmm(total_PC3~Ecomorph+spec_mean_mPC1+within_spec_mPC1+spec_mean_mPC2+within_spec_mPC2,
    random=~phylo+species,family="gaussian",
    ginverse=list(phylo=inv.phylo$Ainv),prior=prior2,data=data.2,
    nitt=1000000,burnin=1000,thin=500,scale=TRUE,verbose=FALSE)
  summary(model_kPC3)
```

```
## 
##  Iterations = 1001:999501
##  Thinning interval  = 500
##  Sample size  = 1998 
## 
##  DIC: 133.1827 
## 
##  G-structure:  ~phylo
## 
##       post.mean l-95% CI u-95% CI eff.samp
## phylo      2.23 0.002837    9.721     1772
## 
##                ~species
## 
##         post.mean l-95% CI u-95% CI eff.samp
## species    0.2064 0.002043    0.739     1998
## 
##  R-structure:  ~units
## 
##       post.mean l-95% CI u-95% CI eff.samp
## units    0.7289   0.4159    1.069     1998
## 
##  Location effects: total_PC3 ~ Ecomorph + spec_mean_mPC1 + within_spec_mPC1 + spec_mean_mPC2 + within_spec_mPC2 
## 
##                    post.mean  l-95% CI  u-95% CI eff.samp   pMCMC   
## (Intercept)         0.717801 -0.242762  1.788110     1998 0.13313   
## EcomorphFlower      0.510345 -1.204356  2.633953     1998 0.49950   
## EcomorphGeneralist -0.512639 -1.954029  0.923155     1998 0.39339   
## EcomorphStick      -3.327321 -5.408817 -1.160629     1998 0.00901 **
## spec_mean_mPC1      0.887135  0.329436  1.454632     1998 0.01001 * 
## within_spec_mPC1   -0.471008 -1.543502  0.699852     2139 0.41141   
## spec_mean_mPC2      1.418108  0.559725  2.199463     1630 0.00601 **
## within_spec_mPC2    0.005678 -0.869640  0.897629     2134 0.98599   
## ---
## Signif. codes:  0 '***' 0.001 '**' 0.01 '*' 0.05 '.' 0.1 ' ' 1
```

```
m<-emmeans(model_kPC3,specs=~Ecomorph,data=data.2)
summary(m,freq=TRUE)
```

```
##  Ecomorph   emmean    SE  df asymp.LCL asymp.UCL
##  Dead Leaf   0.678 0.527 Inf    -0.354     1.710
##  Flower      1.189 0.764 Inf    -0.309     2.687
##  Generalist  0.166 0.523 Inf    -0.859     1.190
##  Stick      -2.649 1.019 Inf    -4.646    -0.652
## 
## Confidence level used: 0.95
```

```
summary(pairs(m),freq=TRUE)
```

```
##  contrast               estimate    SE  df z.ratio p.value
##  Dead Leaf - Flower       -0.510 0.933 Inf  -0.547  0.9474
##  Dead Leaf - Generalist    0.513 0.753 Inf   0.681  0.9044
##  Dead Leaf - Stick         3.327 1.117 Inf   2.978  0.0154
##  Flower - Generalist       1.023 1.029 Inf   0.994  0.7527
##  Flower - Stick            3.838 1.640 Inf   2.340  0.0892
##  Generalist - Stick        2.815 1.016 Inf   2.771  0.0285
## 
## P value adjustment: tukey method for comparing a family of 4 estimates
```

```
lambda <- model_kPC3$VCV[,'phylo']/
 (model_kPC3$VCV[,'phylo']+model_kPC3$VCV[,'species']+
 model_kPC3$VCV[,'units'])
#lambda Full model
mean(lambda)
```

```
## [1] 0.3834413
```

```
model_kPC3.1<-MCMCglmm(total_PC3~Ecomorph,
    random=~phylo+species,family="gaussian",
    ginverse=list(phylo=inv.phylo$Ainv),prior=prior2,data=data.2,
    nitt=1000000,burnin=1000,thin=500,scale=TRUE,verbose=FALSE)
  summary(model_kPC3.1)
```

```
## 
##  Iterations = 1001:999501
##  Thinning interval  = 500
##  Sample size  = 1998 
## 
##  DIC: 132.8435 
## 
##  G-structure:  ~phylo
## 
##       post.mean l-95% CI u-95% CI eff.samp
## phylo     13.16 0.002554    53.52     1662
## 
##                ~species
## 
##         post.mean l-95% CI u-95% CI eff.samp
## species     1.046 0.003214    3.167     1719
## 
##  R-structure:  ~units
## 
##       post.mean l-95% CI u-95% CI eff.samp
## units    0.7357   0.4331    1.104     1998
## 
##  Location effects: total_PC3 ~ Ecomorph 
## 
##                    post.mean l-95% CI u-95% CI eff.samp pMCMC
## (Intercept)           0.7322  -1.2497   2.8301     1998 0.397
## EcomorphFlower       -1.6789  -4.3966   1.3086     1998 0.182
## EcomorphGeneralist   -0.1844  -2.9541   2.4899     1998 0.865
## EcomorphStick        -0.3156  -3.2540   2.4052     2032 0.798
```

```
m<-emmeans(model_kPC3.1,specs=~Ecomorph,data=data.2)
summary(m,freq=TRUE)
```

```
##  Ecomorph   emmean    SE  df asymp.LCL asymp.UCL
##  Dead Leaf   0.732 1.045 Inf     -1.32     2.780
##  Flower     -0.947 0.969 Inf     -2.85     0.952
##  Generalist  0.548 0.931 Inf     -1.28     2.373
##  Stick       0.417 1.038 Inf     -1.62     2.450
## 
## Confidence level used: 0.95
```

```
summary(pairs(m),freq=TRUE)
```

```
##  contrast               estimate   SE  df z.ratio p.value
##  Dead Leaf - Flower        1.679 1.40 Inf   1.201  0.6262
##  Dead Leaf - Generalist    0.184 1.36 Inf   0.136  0.9991
##  Dead Leaf - Stick         0.316 1.44 Inf   0.219  0.9963
##  Flower - Generalist      -1.494 1.36 Inf  -1.098  0.6908
##  Flower - Stick           -1.363 1.41 Inf  -0.964  0.7700
##  Generalist - Stick        0.131 1.25 Inf   0.105  0.9996
## 
## P value adjustment: tukey method for comparing a family of 4 estimates
```

```
lambda <- model_kPC3.1$VCV[,'phylo']/
 (model_kPC3.1$VCV[,'phylo']+model_kPC3.1$VCV[,'species']+
 model_kPC3.1$VCV[,'units'])
#lambda camouflage only
mean(lambda)
```

```
## [1] 0.5552145
```

#### AICc comparison for K PC3 models

```
AICc(model_kPC3,model_kPC3.1)
```

```
##              df     AICc
## model_kPC3   11 151.3790
## model_kPC3.1  7 139.2445
```

# Figures

## Data preparation

```
#setting color
dcol<-hcl.colors(4, palette="OrRd")   #dead leaf mimics, to make transparent add alpha=0.4
fcol<-hcl.colors(7, palette="Purp") #flower mimics
scol<-hcl.colors(5, palette="BrwnYl") #stick mimics
gcol<-hcl.colors(7, palette="Emrld")  #generalists
len<-nrow(max.data)

max.data$col<-NA
for (i in 1:len){
    if(max.data$Species[i]=="Deroplatys truncata"){max.data$col[i]<-dcol[2]}
    if(max.data$Species[i]=="Euchomenella heteroptera"){max.data$col[i]<-scol[1]}
    if(max.data$Species[i]=="Hymenopus coronatus"){max.data$col[i]<-fcol[1]}
    if(max.data$Species[i]=="Phyllocrania paradoxa"){max.data$col[i]<-dcol[1]}
    if(max.data$Species[i]=="Pseudocreobotra wahlbergii"){max.data$col[i]<-fcol[3]}
    if(max.data$Species[i]=="Pseudovates chlorophea"){max.data$col[i]<-scol[3]}
    if(max.data$Species[i]=="Theopropus elegans"){max.data$col[i]<-fcol[5]}
    if(max.data$Species[i]=="Stagmomantis limbata"){max.data$col[i]<-gcol[1]}
    if(max.data$Species[i]=="Tenodera sinensis"){max.data$col[i]<-gcol[3]}
  if(max.data$Species[i]=="Chopardiella pouliani"){max.data$col[i]<-gcol[5]}
}   
#this isn't working now?
#setting shape
max.data$sh<-NA
for (i in 1:len){
    if(max.data$Species[i]=="Deroplatys truncata"){max.data$sh[i]<-15}
    if(max.data$Species[i]=="Euchomenella heteroptera"){max.data$sh[i]<-17}
    if(max.data$Species[i]=="Hymenopus coronatus"){max.data$sh[i]<-18}
    if(max.data$Species[i]=="Phyllocrania paradoxa"){max.data$sh[i]<-15}
    if(max.data$Species[i]=="Pseudocreobotra wahlbergii"){max.data$sh[i]<-18}
    if(max.data$Species[i]=="Pseudovates chlorophea"){max.data$sh[i]<-17}
    if(max.data$Species[i]=="Theopropus elegans"){max.data$sh[i]<-18}
    if(max.data$Species[i]=="Stagmomantis limbata"){max.data$sh[i]<-16}
    if(max.data$Species[i]=="Tenodera sinensis"){max.data$sh[i]<-16}
  if(max.data$Species[i]=="Chopardiella pouliani"){max.data$sh[i]<-16}
}   

data.1<-aggregate(max.data[,16:100], by=list(Tip.label=max.data$Phylo,max.data$Species,max.data$Ecomorph),
FUN=mean, na.rm=TRUE) 
colnames(data.1)[2] ="Species"
colnames(data.1)[3] ="Ecomorph"


data.1$Species <- trimws(data.1$Species)
dcol<-hcl.colors(4, palette="OrRd")   #dead leaf mimics
fcol<-hcl.colors(7, palette="Purp") #flower mimics
scol<-hcl.colors(5, palette="BrwnYl") #stick mimics
gcol<-hcl.colors(7, palette="Emrld")  #generalists
len<-nrow(data.1)

data.1$col<-NA
for (i in 1:len){
    if(data.1$Species[i]=="Deroplatys truncata"){data.1$col[i]<-dcol[2]}
    if(data.1$Species[i]=="Euchomenella heteroptera"){data.1$col[i]<-scol[1]}
    if(data.1$Species[i]=="Hymenopus coronatus"){data.1$col[i]<-fcol[1]}
    if(data.1$Species[i]=="Phyllocrania paradoxa"){data.1$col[i]<-dcol[1]}
    if(data.1$Species[i]=="Pseudocreobotra wahlbergii"){data.1$col[i]<-fcol[3]}
    if(data.1$Species[i]=="Pseudovates chlorophea"){data.1$col[i]<-scol[3]}
    if(data.1$Species[i]=="Theopropus elegans"){data.1$col[i]<-fcol[5]}
    if(data.1$Species[i]=="Stagmomantis limbata"){data.1$col[i]<-gcol[1]}
    if(data.1$Species[i]=="Tenodera sinensis"){data.1$col[i]<-gcol[3]}
  if(data.1$Species[i]=="Chopardiella pouliani"){data.1$col[i]<-gcol[5]}
}   

#setting shape
data.1$sh<-NA
for (i in 1:len){
    if(data.1$Species[i]=="Deroplatys truncata"){data.1$sh[i]<-22}
    if(data.1$Species[i]=="Euchomenella heteroptera"){data.1$sh[i]<-24}
    if(data.1$Species[i]=="Hymenopus coronatus"){data.1$sh[i]<-23}
    if(data.1$Species[i]=="Phyllocrania paradoxa"){data.1$sh[i]<-22}
    if(data.1$Species[i]=="Pseudocreobotra wahlbergii"){data.1$sh[i]<-23}
    if(data.1$Species[i]=="Pseudovates chlorophea"){data.1$sh[i]<-24}
    if(data.1$Species[i]=="Theopropus elegans"){data.1$sh[i]<-23}
    if(data.1$Species[i]=="Stagmomantis limbata"){data.1$sh[i]<-21}
    if(data.1$Species[i]=="Tenodera sinensis"){data.1$sh[i]<-21}
  if(data.1$Species[i]=="Chopardiella pouliani"){data.1$sh[i]<-21}
}   

dcol<-hcl.colors(4, palette="OrRd",alpha=0.4)   #dead leaf mimics
fcol<-hcl.colors(7, palette="Purp",alpha=0.4) #flower mimics
scol<-hcl.colors(5, palette="BrwnYl",alpha=0.4) #stick mimics
gcol<-hcl.colors(7, palette="Emrld",alpha=0.4)  #generalists
len<-nrow(data.2)
data.2$species <- trimws(data.2$species)
data.2$col<-NA
for (i in 1:len){
    if(data.2$species[i]=="Deroplatys truncata"){data.2$col[i]<-dcol[2]}
    if(data.2$species[i]=="Euchomenella heteroptera"){data.2$col[i]<-scol[1]}
    if(data.2$species[i]=="Hymenopus coronatus"){data.2$col[i]<-fcol[1]}
    if(data.2$species[i]=="Phyllocrania paradoxa"){data.2$col[i]<-dcol[1]}
    if(data.2$species[i]=="Pseudocreobotra wahlbergii"){data.2$col[i]<-fcol[3]}
    if(data.2$species[i]=="Pseudovates chlorophea"){data.2$col[i]<-scol[3]}
    if(data.2$species[i]=="Theopropus elegans"){data.2$col[i]<-fcol[5]}
    if(data.2$species[i]=="Stagmomantis limbata"){data.2$col[i]<-gcol[1]}
    if(data.2$species[i]=="Tenodera sinensis"){data.2$col[i]<-gcol[3]}
  if(data.2$species[i]=="Chopardiella pouliani"){data.2$col[i]<-gcol[5]}
}   

#setting shape
data.2$sh<-NA
for (i in 1:len){
    if(data.2$species[i]=="Deroplatys truncata"){data.2$sh[i]<-22}
    if(data.2$species[i]=="Euchomenella heteroptera"){data.2$sh[i]<-24}
    if(data.2$species[i]=="Hymenopus coronatus"){data.2$sh[i]<-23}
    if(data.2$species[i]=="Phyllocrania paradoxa"){data.2$sh[i]<-22}
    if(data.2$species[i]=="Pseudocreobotra wahlbergii"){data.2$sh[i]<-23}
    if(data.2$species[i]=="Pseudovates chlorophea"){data.2$sh[i]<-24}
    if(data.2$species[i]=="Theopropus elegans"){data.2$sh[i]<-23}
    if(data.2$species[i]=="Stagmomantis limbata"){data.2$sh[i]<-21}
    if(data.2$species[i]=="Tenodera sinensis"){data.2$sh[i]<-21}
  if(data.2$species[i]=="Chopardiella pouliani"){data.2$sh[i]<-21}
}
```

## Figure 1: Phylogeny

```
tree.1$tip.label[match(data.1$Tip.label, tree.1$tip.label)] <- data.1$Species
rownames(data.1)<-data.1$Species
is_tip <- tree.1$edge[,2] <= length(tree.1$tip.label)
ordered_tips <- tree.1$edge[is_tip, 2]
tree.1$tip.label[ordered_tips]
```

```
##  [1] "Phyllocrania paradoxa"      "Hymenopus coronatus"       
##  [3] "Theopropus elegans"         "Pseudocreobotra wahlbergii"
##  [5] "Euchomenella heteroptera"   "Deroplatys truncata"       
##  [7] "Tenodera sinensis"          "Stagmomantis limbata"      
##  [9] "Chopardiella pouliani"      "Pseudovates chlorophea"
```

```
data.1<-data.1[tree.1$tip.label,]
attach(data.1)
```

```
## The following objects are masked from data.1 (pos = 3):
## 
##     Approach.Time, AT_COA, AT_FEA, AT_MnTIBAV, AT_MxCOAV, AT_MxFEAV,
##     AT_MxTIBAV, AT_TIBA, B_Tibia_angle, BD_app, BD_dist_cm, BD_Swp,
##     BD_veloc_cm.s, Body_length, CF_lateral_disp, Coxa_angle, Coxa_AV,
##     Coxa_dist, Coxa_length, Coxa_LV, Coxa.start, Femur_angle, Femur_AV,
##     Femur_dist, Femur_Length, Femur_LV, Femur.start, FT_lateral_disp,
##     IJC.cf.mn, IJC.cf.R2, IJC.cf.sd, IJC.cf.sl, IJC.ct.mn, IJC.ct.R2,
##     IJC.ct.sd, IJC.ct.sl, IJC.ft.mn, IJC.ft.R2, IJC.ft.sd, IJC.ft.sl,
##     Max..Volume, Percent_lunge, Percent.Arm, PP_angle_at, PP_angle_st,
##     PP_angle_ttpc, PP_dist_cm_at, PP_dist_cm_st, PP_dist_cm_ttpc,
##     PPD_m_app, PPD_m_swp, ST_Mn_TIBAV, ST_MxCOAV, ST_MxFEAV,
##     ST_MxTIBAV, TIB_AV_max, tib_lateral_disp, Tib.start, TIB.SYNC,
##     Tibia_angle, Tibia_AV_min, Tibia_dist, Tibia_expansion,
##     Tibia_expansion_velocity_max, Tibia_expansion_velocity_min,
##     Tibia_length, Tibia_LV_max, Tibia_LV_min, Tip.label, TTP_B.TIBA,
##     TTP_COA, TTP_COD, TTP_FEA, TTP_FED, TTP_TIBA, TTP_TIBD, TTP_TIBE,
##     TTPC.Approach, TTPC.Sweep
```

```
name.check(tree.1, data.1)
```

```
## [1] "OK"
```

```
#to save use code below
#png(file="Mantis functional phylogeny 1.png", height=18, width=20, units="in",res=300)
#pdf(file="Mantid functional phylogeny.pdf", height=10, width=20)

#quartz(width=20, height=10)
par(mar=c(5.1,8,4.1,2.1))
plot(tree.1,edge.width=4,align.tip.label=FALSE,label.offset=0.005,cex=2, tip.color=data.1$col)
#axisPhylo()
tiplabels(tip=c(1:length(tree.1$tip.label)), pch=data.1$sh, bg=data.1$col,cex=4)
points(0,10,pch=21,bg="black",cex=3)
text(0.002,10,"Generalists",cex=1.5,pos=4)
points(0,9.6,pch=22,bg="black",cex=3)
text(0.002,9.6,"Dead Leaf",cex=1.5,pos=4)
points(0,9.2,pch=23,bg="black",cex=3)
text(0.002,9.2,"Flower",cex=1.5,pos=4)
points(0,8.8,pch=24,bg="black",cex=3)
text(0.002,8.8,"Stick",cex=1.5,pos=4)
text(0.02,7.7, "Mantidae",cex=1.2)
text(0.006,5.3, "Deroplatyidae",cex=1.2)
text(0.0018,1.9, "Hymenopodidae",cex=1.2)
```

```
#dev.off()
```

## Figure 2: Morphospace

```
X<-cbind(data.1$mPC1,data.1$mPC2)
  row.names(X)<-data.1$Species

  #to save use code below
#png(file="Ecomorph Morpho Space.png", height=12, width=14, units="in",res=300)
par(mar=c(5.1,6,4.1,6.5))
plot(data.1$mPC1,data.1$mPC2,col="white",xlim=c(-4,4),ylim=c(-3,3),xlab="Morphology PC1 (68%)",ylab="Morphology PC2 (28%)",cex.lab=2,cex.axis=1.5)
phylomorphospace(tree.1,X[,1:2],lwd=3,label="off",node.size=0,add=TRUE)
points(data.2$mPC1,data.2$mPC2,pch=data.2$sh,bg=data.2$col,cex=4)
points(data.1$mPC1,data.1$mPC2,pch=data.1$sh,bg=data.1$col,cex=6)
mtext("Smaller",side=1,at=-4,line=2.5,cex=1.5)
mtext("Shorter coxa & femur",side=1,at=-4,line=3.5,cex=1.5)
mtext("Larger ",side=1,at=4,line=2.5,cex=1.5)
mtext("Longer coxa & femur",side=1,at=4,line=3.5,cex=1.5)
mtext("Smaller",side=2,at=-2.5,line=3.5,cex=1.5)
mtext("longer tibia",side=2,at=-2.5,line=2.5,cex=1.5)
mtext("Larger",side=2,at=2.5,line=3.5,cex=1.5)
mtext("shorter tibia",side=2,at=2.5,line=2.5,cex=1.5)
```

```
#dev.off()
```

## Figure 3: Kinematic PC 1 and 2 with biplot

```
layout_mat<-matrix(c(1,2,3,4,5,4), 2, 3, byrow = FALSE)
#layout_mat
my_layout<-layout(mat=layout_mat,widths=c(6,2,2), heights=c(4,4),respect=TRUE)
#layout.show(my_layout)

#to save use code below
png(file="Fig. 3 Ecomorph Functional Space loadings.png", height=8, width=10, units="in",res=300)
my_layout<-layout(mat=layout_mat,widths=c(6,2,2), heights=c(4,4),respect=TRUE)
par(mar = c(5.1, 5.1, 0, 2))
plot(max.data$total_PC1,max.data$total_PC2,pch=max.data$sh, cex=3,cex.lab=2,cex.axis=1.5,col=max.data$col,xlim=c(-4,4),ylim=c(-3,3),xlab="Kinematic PC1 (25.91%)",ylab="Kinematic PC2 (16.18%)",axes=FALSE)
text(-4,3,"A",cex=3)
axis(1)
axis(2)
#2 phylomorphospace
X<-cbind(data.1$total_PC1,data.1$total_PC2)
  row.names(X)<-data.1$Species
par(mar=c(5.1,5.1,0,2))
plot(data.1$total_PC1,data.1$total_PC2,col="white",xlim=c(-4,4),ylim=c(-3,3),xlab="Kinematic PC1 (25.91%)",ylab="Kinematic PC2 (16.18%)",cex.lab=2,cex.axis=1.5,axes=FALSE)
phylomorphospace(tree.1,X[,1:2],lwd=3,label="off",node.size=0,add=TRUE)
points(data.2$total_PC1,data.2$total_PC2,pch=data.2$sh,bg=data.2$col,cex=4)
points(data.1$total_PC1,data.1$total_PC2,pch=data.1$sh,bg=data.1$col,cex=6)
text(-4,3,"B",cex=3)
axis(1)
axis(2)
#3 legend 1
x<-c(1,10)
y<-c(1,10)
par(mar = c(0, 0, 0, 0))
plot(x,y,col="white",xlim=c(-4,4),ylim=c(-3,3),xlab="",ylab="",axes=FALSE)
unique_Species <- unique(max.data$Species)
legend_label <- sapply(unique_Species, function(label) {
  as.expression(substitute(italic(label), list(label=label)))
})

legend("center", 
       legend = unique_Species,
       pch = c(16, 16, 15, 17, 18, 17, 15, 18, 18, 16),
       title = "Species", 
       col = unique(max.data$col), pt.cex = 1,cex=1)


#4 plot of just the loadings
par(mar = c(5.1, 1, 1, 0))
plot(pca_total$loadings[,1],pca_total$loadings[,2], xlim=c(-0.5,0.6),ylim=c(-0.2,0.6),col="white",axes=FALSE,ylab="",xlab="",main="Kinematic Contributions")
axis(1)
axis(2)
 arrows(x0 = 0, x1 = pca_total$loadings[,1], 
       y0 = 0, y1 = pca_total$loadings[,2], 
       col = "red", 
       length = 0.08, 
       lwd = 1,
       angle = 30)       
labels<-c("Coxa LV","Femur LV","Coxa AV","Femur AV","Tibia AV", "Tibia LV","Coxa SA","Approach","PP Angle","PP Dist","BD Vel","BD Disp","Sweep", "CF Lat Disp", "FT Lat Disp","Tib Lat Disp")
text(x = pca_total$loadings[,1], y = pca_total$loadings[,2], 
     labels = labels, 
     cex = 1,
     font = 2,
     col = "gray10", 
     pos = c(4, 3, 2, 1, 3, 1))  
 text(-0.4,0.6,"C",cex=3)     
#5 second legend      
par(mar = c(0, 0, 0, 0))
plot(x,y,col="white",xlim=c(-4,4),ylim=c(-3,3),xlab="",ylab="",axes=FALSE)
legend("center", 
       legend = unique(max.data$Ecomorph), 
       pch = unique(max.data$sh), cex=1,pt.cex=1)

dev.off()
```

```
## quartz_off_screen 
##                 2
```

## Figure 4: Kinematic PC 1 and 3 with biplot

```
layout_mat<-matrix(c(1,2,3,4,5,4), 2, 3, byrow = FALSE)
#layout_mat
my_layout<-layout(mat=layout_mat,widths=c(6,2,2), heights=c(4,4),respect=TRUE)
#layout.show(my_layout)

#to save use code below
png(file="Fig. 4 Ecomorph Functional Space loadings.png", height=8, width=10, units="in",res=300)
my_layout<-layout(mat=layout_mat,widths=c(6,2,2), heights=c(4,4),respect=TRUE)
par(mar = c(5.1, 5.1, 0, 2))
#1 all max attempts
plot(max.data$total_PC1,max.data$total_PC3,pch=max.data$sh, cex=3,cex.lab=2,cex.axis=1.5,col=max.data$col,xlim=c(-4,4),ylim=c(-4,4),xlab="Kinematic PC1 (25.91%)",ylab="Kinematic PC3 (15.04%)",axes=FALSE)
text(-4,4,"A",cex=3)
axis(1)
axis(2)

#2 phylospace
X<-cbind(data.1$total_PC1,data.1$total_PC3)
  row.names(X)<-data.1$Species
par(mar=c(5.1,5.1,0,2))
plot(data.1$total_PC1,data.1$total_PC3,col="white",xlim=c(-4,4),ylim=c(-4,4),xlab="Kinematic PC1 (25.91%)",ylab="Kinematic PC3 (15.04%)",cex.lab=2,cex.axis=1.5,axes=FALSE)
phylomorphospace(tree.1,X[,1:2],lwd=3,label="off",node.size=0,add=TRUE)
points(data.2$total_PC1,data.2$total_PC3,pch=data.2$sh,bg=data.2$col,cex=4)
points(data.1$total_PC1,data.1$total_PC3,pch=data.1$sh,bg=data.1$col,cex=6)
text(-4,4,"B",cex=3)
axis(1)
axis(2)

#3 legend 1
x<-c(1,10)
y<-c(1,10)
par(mar = c(0, 0, 0, 0))
plot(x,y,col="white",xlim=c(-4,4),ylim=c(-3,3),xlab="",ylab="",axes=FALSE)
unique_Species <- unique(max.data$Species)
legend_label <- sapply(unique_Species, function(label) {
  as.expression(substitute(italic(label), list(label=label)))
})

legend("center", 
       legend = unique_Species,
       pch = c(16, 16, 15, 17, 18, 17, 15, 18, 18, 16),
       title = "Species", 
       col = unique(max.data$col), pt.cex = 1,cex=1)


#3 plot of just the loadings
par(mar = c(5.1, 1, 1, 0))
plot(pca_total$loadings[,1],pca_total$loadings[,3], xlim=c(-0.5,0.6),ylim=c(-0.6,0.4),col="white",axes=FALSE,ylab="",xlab="",main="Kinematic Contributions")
axis(1)
axis(2)
 arrows(x0 = 0, x1 = pca_total$loadings[,1], 
       y0 = 0, y1 = pca_total$loadings[,3], 
       col = "red", 
       length = 0.08, 
       lwd = 1,
       angle = 30)       
labels<-c("Coxa LV","Femur LV","Coxa AV","Femur AV","Tibia AV", "Tibia LV","Coxa SA","Approach","PP Angle","PP Dist","BD Vel","BD Disp","Sweep", "CF Lat Disp", "FT Lat Disp","Tib Lat Disp")
text(x = pca_total$loadings[,1], y = pca_total$loadings[,3], 
     labels = labels, 
     cex = 1,
     font = 2,
     col = "gray10", 
     pos = c(4, 3, 2, 1, 3, 1))  
 text(-0.4,0.4,"C",cex=3)    
#4 second legend      
par(mar = c(0, 0, 0, 0))
plot(x,y,col="white",xlim=c(-4,4),ylim=c(-3,3),xlab="",ylab="",axes=FALSE)
legend("center", 
       legend = unique(max.data$Ecomorph), 
       pch = unique(max.data$sh), cex=1,pt.cex=1)

dev.off()
```

```
## quartz_off_screen 
##                 2
```

## Figure 5: Coxa start differences

```
#assign tip orders for y axis
len<-nrow(data.2)

for (i in 1:len){
    if(data.2$species[i]=="Deroplatys truncata"){data.2$tip.order[i]<-6}
    if(data.2$species[i]=="Euchomenella heteroptera"){data.2$tip.order[i]<-5}
    if(data.2$species[i]=="Hymenopus coronatus"){data.2$tip.order[i]<-2}
    if(data.2$species[i]=="Phyllocrania paradoxa"){data.2$tip.order[i]<-1}
    if(data.2$species[i]=="Pseudocreobotra wahlbergii"){data.2$tip.order[i]<-4}
    if(data.2$species[i]=="Pseudovates chlorophea"){data.2$tip.order[i]<-10}
    if(data.2$species[i]=="Theopropus elegans"){data.2$tip.order[i]<-3}
    if(data.2$species[i]=="Stagmomantis limbata"){data.2$tip.order[i]<-8}
    if(data.2$species[i]=="Tenodera sinensis"){data.2$tip.order[i]<-7}
  if(data.2$species[i]=="Chopardiella pouliani"){data.2$tip.order[i]<-9}
}   

len<-nrow(data.1)

for (i in 1:len){
    if(data.1$Species[i]=="Deroplatys truncata"){data.1$tip.order[i]<-6}
    if(data.1$Species[i]=="Euchomenella heteroptera"){data.1$tip.order[i]<-5}
    if(data.1$Species[i]=="Hymenopus coronatus"){data.1$tip.order[i]<-2}
    if(data.1$Species[i]=="Phyllocrania paradoxa"){data.1$tip.order[i]<-1}
    if(data.1$Species[i]=="Pseudocreobotra wahlbergii"){data.1$tip.order[i]<-4}
    if(data.1$Species[i]=="Pseudovates chlorophea"){data.1$tip.order[i]<-10}
    if(data.1$Species[i]=="Theopropus elegans"){data.1$tip.order[i]<-3}
    if(data.1$Species[i]=="Stagmomantis limbata"){data.1$tip.order[i]<-8}
    if(data.1$Species[i]=="Tenodera sinensis"){data.1$tip.order[i]<-7}
  if(data.1$Species[i]=="Chopardiella pouliani"){data.1$tip.order[i]<-9}
}

#use code below to save
#png(file="Fig. 6 coxa start.png", height=10, width=10, units="in",res=300)
layout(matrix(c(1,1,2,2), 2, 2, byrow = FALSE ))
par(mar=c(4, 1, 4, 0))
plot(tree.1,edge.width=4,align.tip.label=FALSE,label.offset=0.01,cex=1.5, tip.color="black",adj=1)
#axisPhylo()
tiplabels(tip=c(1:length(tree.1$tip.label)), pch=data.1$sh, bg=data.1$col,cex=4)
plot(data.1$Coxa.start,data.1$tip.order, pch=data.1$sh,bg=data.1$col,cex=6,xlim=c(10,115),ylab=" ",axes=FALSE,xlab="Coxa Start Angle (degrees)",cex.lab=1.5)
points(data.2$Coxa.start,data.2$tip.order, pch=data.2$sh,bg=data.2$col,cex=2)
axis(side=1,cex.axis=1.5)
abline(v=50,lty="dashed")
mtext("Basigrade", at=20)
mtext("Anterograde", at=80)
```

```
#dev.off()
```

## Figure S3: KPC1 extreme traits

```
layout_mat<-matrix(c(1,1,2,3,4), 1, 5, byrow = FALSE)
#extreme PC axes
#use code below to save
#png(file="Fig. 4 PC1 extremes.png", height=8, width=16, units="in",res=300)
my_layout<-layout(mat=layout_mat,widths=c(3,3,5,5,5), heights=c(10,10),respect=TRUE)
par(mar=c(4, 1, 4, 0))
plot(tree.1,edge.width=4,align.tip.label=FALSE,label.offset=0.01,cex=1.8, tip.color="black",adj=1)
#axisPhylo()
tiplabels(tip=c(1:length(tree.1$tip.label)), pch=data.1$sh, bg=data.1$col,cex=4)
plot(data.1$Femur_AV,data.1$tip.order, pch=data.1$sh,bg=data.1$col,cex=6,xlim=c(2000,11000),ylab=" ",axes=FALSE,xlab=expression(Femur~angular~vel.~(degrees~sec^1)),cex.lab=1.5)
points(data.2$Femur_AV,data.2$tip.order, pch=data.2$sh,bg=data.2$col,cex=2)
axis(side=1,cex.axis=1.5)
segments(11000,0,11000,10)
text(2100,10, "A)",cex=3)
plot(data.1$abs_tibia_AV,data.1$tip.order, pch=data.1$sh,bg=data.1$col,cex=6,xlim=c(6000,17000),ylab=" ",axes=FALSE,xlab=expression(Tibia~angular~vel.~(degrees~sec^1)),cex.lab=1.5)
points(data.2$abs_tibia_AV,data.2$tip.order, pch=data.2$sh,bg=data.2$col,cex=2)
axis(side=1,cex.axis=1.5)
segments(17000,0,17000,10)
text(6000,10, "B)",cex=3)
plot(data.1$BD_dist_cm,data.1$tip.order, pch=data.1$sh,bg=data.1$col,cex=6,xlim=c(0.2,2.5),ylab=" ",axes=FALSE,xlab=expression(Body~Displacement~(cm)),cex.lab=1.5)
points(data.2$BD_dist_cm,data.2$tip.order, pch=data.2$sh,bg=data.2$col,cex=2)
axis(side=1,cex.axis=1.5)
text(0.25,10, "C)",cex=3)
```

```
#dev.off()
```

## Figure S4: KPC2 extreme traits

```
# approach, prey dist coxa and bd lv
#use code below to save
#png(file="Fig. 5 PC2 extremes.png", height=8, width=16, units="in",res=300)
my_layout<-layout(mat=layout_mat,widths=c(3,3,5,5,5), heights=c(10,10),respect=TRUE)
par(mar=c(4, 1, 4, 0))
plot(tree.1,edge.width=4,align.tip.label=FALSE,label.offset=0.01,cex=1.8, tip.color="black",adj=1)
#axisPhylo()
tiplabels(tip=c(1:length(tree.1$tip.label)), pch=data.1$sh, bg=data.1$col,cex=4)
plot(data.1$Approach.Time,data.1$tip.order, pch=data.1$sh,bg=data.1$col,cex=6,xlim=c(0.001,0.06),ylab=" ",axes=FALSE,xlab="Approach Time (sec.)",cex.lab=1.5)
points(data.2$Approach.Time,data.2$tip.order, pch=data.2$sh,bg=data.2$col,cex=2)
axis(side=1,cex.axis=1.5)
segments(0.062,0,0.062,10)
text(0.003,10, "A)",cex=3)
plot(data.1$Coxa_LV,data.1$tip.order, pch=data.1$sh,bg=data.1$col,cex=6,xlim=c(40,170),ylab=" ",axes=FALSE,xlab=expression(Coxa~LV~(cm~s^-1)),cex.lab=1.5)
points(data.2$Coxa_LV,data.2$tip.order, pch=data.2$sh,bg=data.2$col,cex=2)
axis(side=1,cex.axis=1.5)
segments(170,0,170,10)
text(40,10, "B)",cex=3)
plot(data.1$BD_veloc_cm.s,data.1$tip.order, pch=data.1$sh,bg=data.1$col,cex=6,xlim=c(13,90),ylab=" ",axes=FALSE,xlab=expression(Body~Velocity~(cm~s^-1)),cex.lab=1.5)
points(data.2$BD_veloc_cm.s,data.2$tip.order, pch=data.2$sh,bg=data.2$col,cex=2)
axis(side=1,cex.axis=1.5)
text(14,10, "C)",cex=3)
```

```
#dev.off()
```

## Figure S5: morphology vs. kinematics

```
#use code below to save
#png(file="Figure 7.png", height=10, width=16, units="in",res=300)
layout(matrix(c(1,2,1,2), 2, 2, byrow = TRUE ))
X<-cbind(data.1$mPC1,data.1$Coxa_LV)
  row.names(X)<-data.1$Species
par(mar=c(5.1,6,4.1,6.5))
plot(data.1$mPC1,data.1$Coxa_LV,col="white",xlim=c(-4,4),ylim=c(40,170),xlab="Morphology PC1 (68%)",ylab=expression(Coxa~LV~(cm~s^-1)),cex.lab=2,cex.axis=1.5)
phylomorphospace(tree.1,X[,1:2],lwd=3,label="off",node.size=0,add=TRUE)
points(data.2$mPC1,data.2$Coxa_LV,pch=data.2$sh,bg=data.2$col,cex=4)
points(data.1$mPC1,data.1$Coxa_LV,pch=data.1$sh,bg=data.1$col,cex=6)
mtext("Longer coxa & femur",side=1,at=4,line=2.5,cex=1.2)
mtext("A)",side=3,at=-4,line=1,cex=1.5)

X<-cbind(data.1$mPC2,data.1$Coxa.start)
  row.names(X)<-data.1$Species
par(mar=c(5.1,6,4.1,6.5))
plot(data.1$mPC2,data.1$Coxa.start,col="white",xlim=c(-3,3),ylim=c(10,115),xlab="Morphology PC2 (28%)",ylab="Coxa Start Angle (degrees)",cex.lab=2,cex.axis=1.5)
phylomorphospace(tree.1,X[,1:2],lwd=3,label="off",node.size=0,add=TRUE)
points(data.2$mPC2,data.2$Coxa.start,pch=data.2$sh,bg=data.2$col,cex=4)
points(data.1$mPC2,data.1$Coxa.start,pch=data.1$sh,bg=data.1$col,cex=6)
mtext("Longer tibia",side=1,at=-2.5,line=2.5,cex=1.2)
mtext("B)",side=3,at=-3,line=1,cex=1.5)
```

```
#dev.off()
```
